# Activation of proresolving macrophages in dorsal root ganglia attenuates persistent arthritis pain

**DOI:** 10.1073/pnas.2416343122

**Published:** 2025-03-10

**Authors:** Silvia Oggero, Mathieu-Benoit Voisin, Francesca Picco, Miguel Á. Huerta, Chiara Cecconello, Thomas Burgoyne, Mauro Perretti, Marzia Malcangio

**Affiliations:** ^a^Sensory, Pain and Regeneration Centre, Institute of Psychiatry, Psychology and Neuroscience, Kings College London, Guys’ Campus, London SE1 1UL, United Kingdom; ^b^William Harvey Research Institute, Faculty of Medicine and Dentistry, Queen Mary University of London, London EC1M 6BQ, United Kingdom; ^c^Department of Pharmacology, University of Granada, Granada 18016, Spain; ^d^Institute of Ophthalmology, Faculty of Brain Sciences, University College London, London EC1V 9EL, United Kingdom; ^e^Pediatric Respiratory Medicine, Royal Brompton Hospital, Guy’s and St Thomas’ National Health System Foundation Trust, London SW3 6NP, United Kingdom

**Keywords:** proresolving macrophages, neutrophils, efferocytosis, rheumatoid arthritis, chronic pain

## Abstract

Pain independent of disease activity is frequently reported by rheumatoid arthritis patients and remains undertreated. Preclinical evidence suggests that imbalance of neuroimmune proresolving interactions within dorsal root ganglia (DRG) rather than at the site of inflammation plays mechanistic roles in persistent arthritis pain. Here, we inhibited production of proresolving lipid mediators by silencing 12/15-lipoxygenase expression in CX3CR1^+^ monocyte/macrophages conditional knockout (cKO) mice. In an arthritis model, hind paw mechanical hypersensitivity is exacerbated in male and female cKO mice in association with DRG infiltration of neutrophils, which migrate in response to leukotriene B_4_ released by macrophages through 5-lipoxygenase conversion of arachidonic acid provided by neuron-derived vesicles. Neutrophils apoptosis promotes primary macrophage efferocytosis which is defective in cKO macrophages. In wild-type (WT) and cKO mice, intrathecal injection of MerTK activating antibody, attenuates persistent hypersensitivity and polarizes DRG macrophages toward a proresolving phenotype with production of antinociceptive lipoxin A_4_. Thus, we delineate a neuron–macrophage–neutrophil bidirectional circuit that can be exploited to reduce persistent arthritis pain.

## Significance

Mechanisms of persistent arthritis pain despite resolution of joint inflammation remain undefined. This study indicates that compromised macrophage-driven resolution within dorsal root ganglia (DRG) results in imbalance of proresolving in favor of proinflammatory lipid mediators that sustain nociceptive signaling. Here, we demonstrate that levels of proresolving lipid mediators can be significantly elevated by boosting macrophage polarization toward proresolving phenotype that results in attenuation of persistent inflammatory pain.

Despite pain being a cardinal feature of inflammation that ceases when inflammation resolves, in some circumstances, such as rheumatoid arthritis (RA), pathological pain persists regardless of optimal control of joint pathology (1). People with RA identify pain as the most debilitating symptom, which is only controlled by NSAIDs (1). Thus, there is significant preclinical effort to investigate mechanisms for persistent inflammatory pain aided by the knowledge that the nervous and immune systems play interconnecting roles (2–4). Growing evidence suggests that persistent pain results from a failure of immune cell-mediated proresolving and antinociceptive mechanisms at peripheral and central sites along the pain pathways (5–7). Therefore, the identification of defective endogenous proresolving pathways can provide insights into mechanisms that can be redressed (5). Notably, proresolving approaches aim to activate endogenous reparative processes as opposed to traditional anti-inflammatory strategies that merely block inflammation (8).

In a model of inflammatory arthritis in which pain hypersensitivity persists despite joint swelling resolves, evidence indicates an imbalance of proresolving mediator levels in the dorsal root ganglia (DRG), including resolvin E1 (RvE1), several resolvins of the D-series and Maresin 1 (MaR1) (5).

DRG contain the cell bodies of sensory neurons that innervate the joint and DRG neuron activity engages local macrophages which increase to significant numbers and release pronociceptive factors that lead to persistent inflammatory pain (9). In addition, macrophages can release proresolving lipid mediators through the action of acetylated cyclooxygenase-2 (COX-2) or lipoxygenase (5-LOX, 12/15-LOX) enzymes from diet-derived essential polyunsaturated fatty acids (PUFAs) (10). Types and levels of specific mediators can vary across tissues e.g. blood *versus* a given organ. However, these are consistent with the severity of inflammation and their profile is determined by the cellular environment as biosynthesis is regulated by leukocyte numbers and types, and often requires trans-cellular mechanisms (11, 12).

Acting on specific G-protein-coupled receptors (GPCRs), proresolving lipid mediators promote resolution of pain through molecular and cellular mechanisms that prevail over proinflammatory mediators (7). Proresolving lipid mediators inhibit inflammatory pain at lower doses than morphine and via mechanisms that involve modulation of neurons and immune cells (13). For example, MaR1 is synthesized in macrophages by 12-LOX or 15-LOX (in rodents there is only one enzyme termed Alox15), and dose-dependently decreases allodynia in inflammatory pain models (14). MaR1 is antinociceptive through inhibition of TRPV1 receptor activity in nociceptors (15) and reduction of neutrophil and proinflammatory macrophage recruitment to inflamed skin and DRG (5, 14).

In this study, we used the K/BxN serum transfer (ST) model of inflammatory arthritis pain, in which mice display ankle joint swelling and hind paw mechanical hypersensitivity that peak at day 7 K/BxN-ST; however, while joint swelling resolves by day 25 K/BxN-ST, hypersensitivity persists (5, 9). We observed exacerbation of inflammatory arthritis pain in Cx3cr1^Cre:^Alox15^flox/flox^ mice (cKO) that results from impairment of proresolving macrophage interaction with neurons in DRG. Specifically, we delineate a pathway which starts with nociceptive neuron transfer of extracellular vesicles (EV) containing arachidonic acid (AA) to macrophages. AA is converted by 5-LOX into leukotriene B4 (LTB_4_) that promotes neutrophil infiltration in DRG parenchyma. Both LTB_4_ production and neutrophil infiltration are amplified in cKO because Alox15 loss is balanced out by higher 5-LOX expression. Neutrophil apoptosis promotes macrophage efferocytosis which is defective in cKO macrophages but can be boosted by a MerTK activating antibody that activates the receptor tyrosine kinase MER, and promotes clearance of apoptotic cells and release of proresolving factors (16, 17). Under persistent inflammatory pain conditions, MerTK activating antibody exerts antinociceptive action and polarizes DRG macrophages toward a proresolving phenotype, thus providing proof-of-concept evidence for antinociceptive potential of macrophage manipulation.

## Results

### Silencing of Alox15 in Monocytes/Macrophages Is Associated with Exacerbation of Inflammatory Arthritis Pain.

**Fig. 1.**
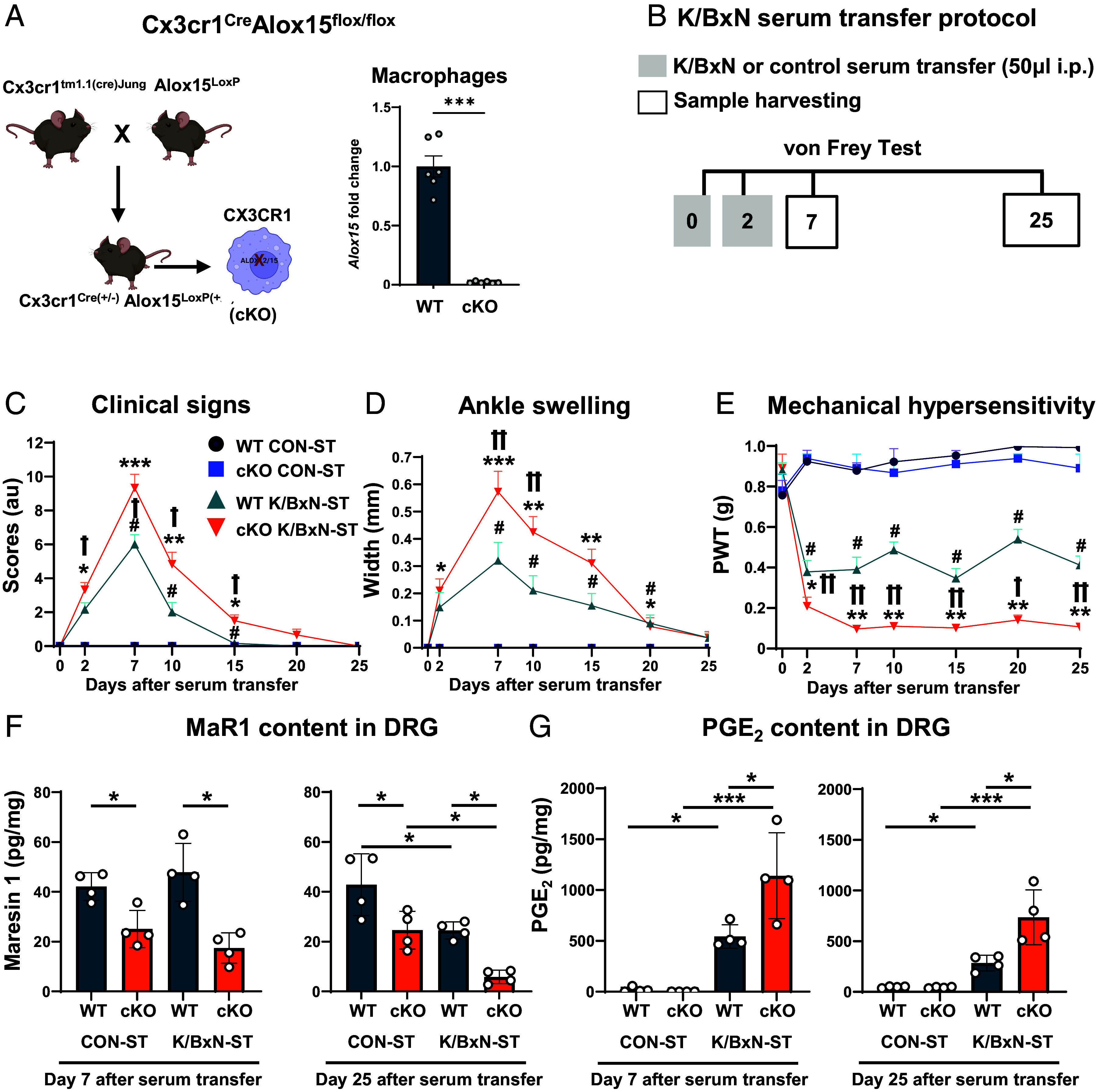
Exacerbation of K/BxN serum transfer associated allodynia in Cx3cr1^cre^Alox15^floxed^ mice. (*A*) Schematic for cx3cr1^Cre^Alox15^loxP/loxP^ (cKO) mice breeding strategy. *Alox15* gene expression quantified as fold change in bone marrow–derived macrophages (BMDMs). A*lox15* absolute expression 0.0246 in WT and 0.0005 in cKO, respectively. (*B*) Diagram for K/BxN and Control (CON) serum transfer (ST) protocol. (*C*) Hind and fore paws clinical scores (arbitrary units), (*D*) ankle swelling, and (*E*) hind paw mechanical hypersensitivity (allodynia, PWT; paw withdrawal thresholds). Data are mean ± SEM, n = 12 mice per group (6 male and 6 female). *P < 0.05, **P < 0.01, ***P < 0.001 cKO K/BxN-ST vs same-day CON-ST; ^#^*P* < 0.05 WT K/BxN-ST vs same-day CON-ST; ^†^*P* < 0.05, ^††^*P* < 0.01 cKO K/BxN-ST vs same-day WT K/BxN-ST, two-way RM ANOVA followed by Tukey’s multiple comparisons test. (*F*) Quantification of Maresin 1 (MaR1) and (*G*) prostaglandin E2 (PGE_2_) contents in DRG. Data are mean ± SEM; n = 4 biological replicates. *P < 0.05, **P < 0.01, ***P < 0.001, one-way ANOVA followed by Bonferroni’s multiple-comparison test.

Considering that monocytes and macrophages are a reliable source of proresolving lipid mediators because they express Alox15, we generated conditional knockout mice (cKO) (Cx3cr1^Cre:^Alox15^flox/flox^) and confirmed silencing of *alox15* in macrophages (Fig. 1*A*).

In wild type (WT) and cKO mice, we assessed development of K/BxN-ST induced inflammatory arthritis (Fig. 1*B*). In line with reduction of Alox15 products with proresolving function, in cKO, we observed that K/BxN-ST was associated with exacerbation of paw swelling (clinical scores and ankle thickness) although, like in WT, swelling peaked at day 7, gradually diminished, and resolved from day 20 onward (Fig. 1 *C* and *D*). Furthermore, mechanical hypersensitivity (allodynia) was more pronounced in cKO than WT, however, in both groups it developed from day 3 onward (Fig. 1*E*), in male and female mice (*SI Appendix*, Fig. S1 *A* and *B*). To further validate our breeding strategy, since Alox15 mediates MaR1 formation (*SI Appendix*, Fig. S1*C*), we quantified DRG levels of MaR1 that has antinociceptive properties. We also quantified levels of prostaglandin E2 (PGE_2_) produced by COX-1 and COX-2 in macrophages (*SI Appendix*, Fig. S1*D*) that has pronociceptive properties. At day 7 after ST, in WT DRG, we found comparable levels of MaR1 in Control ST (CON-ST) and K/BxN-ST whereas contents were lower in cKO DRG, suggesting that Alox15 silencing limits MaR1 formation (Fig. 1*F*). Then, at day 25 after ST, in WT DRG, MaR1 levels were lower in K/BxN-ST than in CON-ST (Fig. 1*F*), confirming proresolving lipid mediator imbalance in concomitance to persistent allodynia. Furthermore, in cKO DRG, MaR1 levels were even lower than in WT in both CON-ST and K/BxN-ST DRG (Fig. 1*F*; day 25), indicating a correlation between reduction of MaR1 levels and exacerbation of allodynia. Concerning PGE_2_, at day 7 after ST, DRG levels of this pronociceptive lipid mediator increased 500-fold in WT and 1,000-fold in cKO DRG compared to CON-ST (Fig. 1*G*) indicating a direct correlation between PGE_2_ levels and day 7-K/BxN-ST (peak) allodynia. Then, at day 25 after ST, PGE_2_ content decreased by half in WT, but to a lesser extent in cKO DRG in concomitance to exacerbation of persistent allodynia in cKO (Fig. 1*G*; day 25).

Thus, cKO data suggest that normally, Alox15 products exert tonic inhibition on mechanisms for paw swelling and mechanical hypersensitivity in inflammatory arthritis, both of which are driven by proinflammatory/nociceptive lipid mediators such as PGE_2_.

Next, considering that in the K/BxN-ST model, monocytes/macrophages infiltrate the inflamed hind paws and accumulate in DRG in response to neuronal activity (9), we evaluated leukocytes phenotype and trafficking at both sites in cKO mice.

### Silencing of Alox15 in Monocytes/Macrophages Is Associated with Neutrophil Infiltration in DRG at Peak Pain and Less Efferocytic Macrophages under Persistent Pain Conditions.

**Fig. 2.**
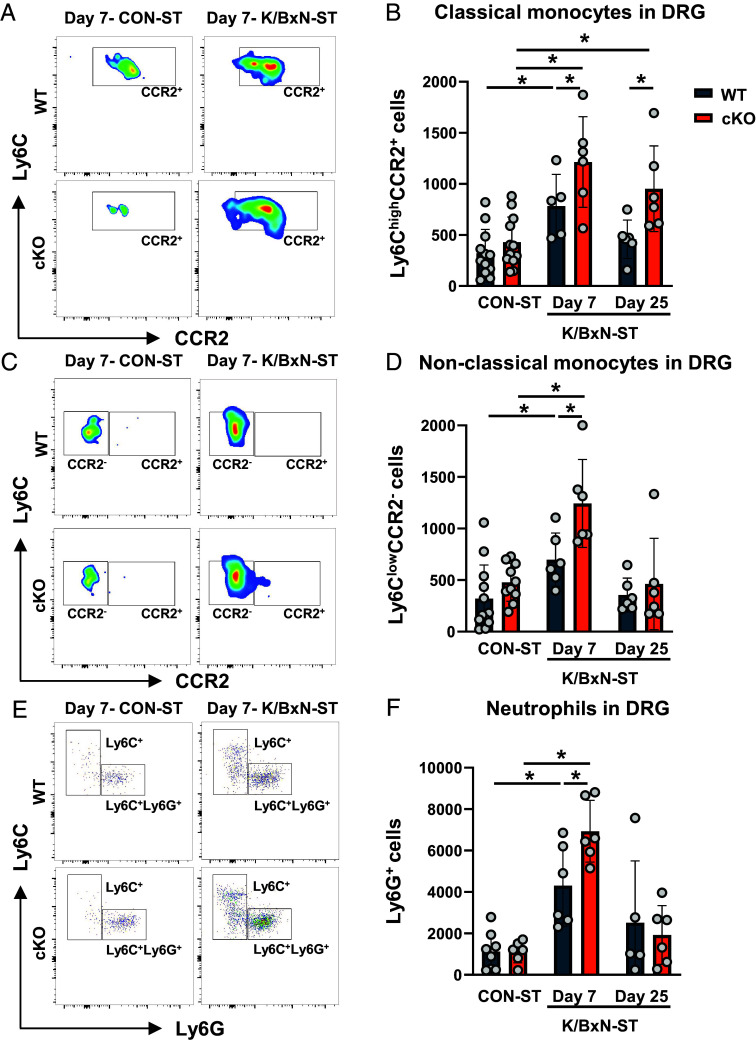
Larger infiltration of neutrophils and monocytes in Cx3cr1^cre^Alox15^floxed^ DRG at day 7 after K/BxN-ST. (*A*) Representative scatter plots of CCR2 receptor expression in L3–L5 DRG CD11b^+^CD45^+^F4/80^−^Ly6C^high^CD43^−^ classical monocytes. (*B*) Quantification of L3–L5 DRG classical monocyte absolute numbers. (*C*) Representative scatter plots of CCR2 receptor expression in L3–L5 DRG CD11b^+^CD45^+^F4/80^−^Ly6C^low^CD43^+^ nonclassical monocytes. (*D*) Quantification of nonclassical monocyte absolute numbers. (*E*) Representative scatter plots of neutrophil Ly6G expression in L3–L5 DRG CD11b^+^CD45^+^F4/80^−^Ly6C^low^CD43^−^ cells. (*F*) Quantification of L3–L5 DRG neutrophil absolute numbers. Data are mean ± SEM. n = 6 to 12 biological replicates. *P < 0.05, one-way ANOVA followed by Tukey’s multiple-comparison test.

Since monocytes populate the DRG in concomitance to peak allodynia (day 7-K/BxN-ST) and M1-like macrophages accumulate in association with persistent allodynia (day 25-K/BxN-ST) (9), we evaluated whether cKO monocyte/macrophage trafficking and phenotypes were distinguishable from WT (flow cytometry gating strategy in *SI Appendix*, Fig. S2 *A* and *B*). At peak hypersensitivity, in male and female cKO DRG both classical (Ly6C^high^F/4/80^−^Ly6G^−^CD43^−^CCR2^+^ cells) and nonclassical (Ly6C^low^CD43^+^CCR2^−^) monocytes (Fig. 2 *A*–*D*) as well as neutrophils (Ly6G^+^cells) (Fig. 2 *E* and *F*) were more numerous than in WT. Notably, classical monocytes (CCR2^+^ cells) were still present in cKO but not WT DRG at day 25-KBxN-ST (Fig. 2 *A* and *B*), suggesting cKO monocytes were still infiltrating at this later time point.

**Fig. 3.**
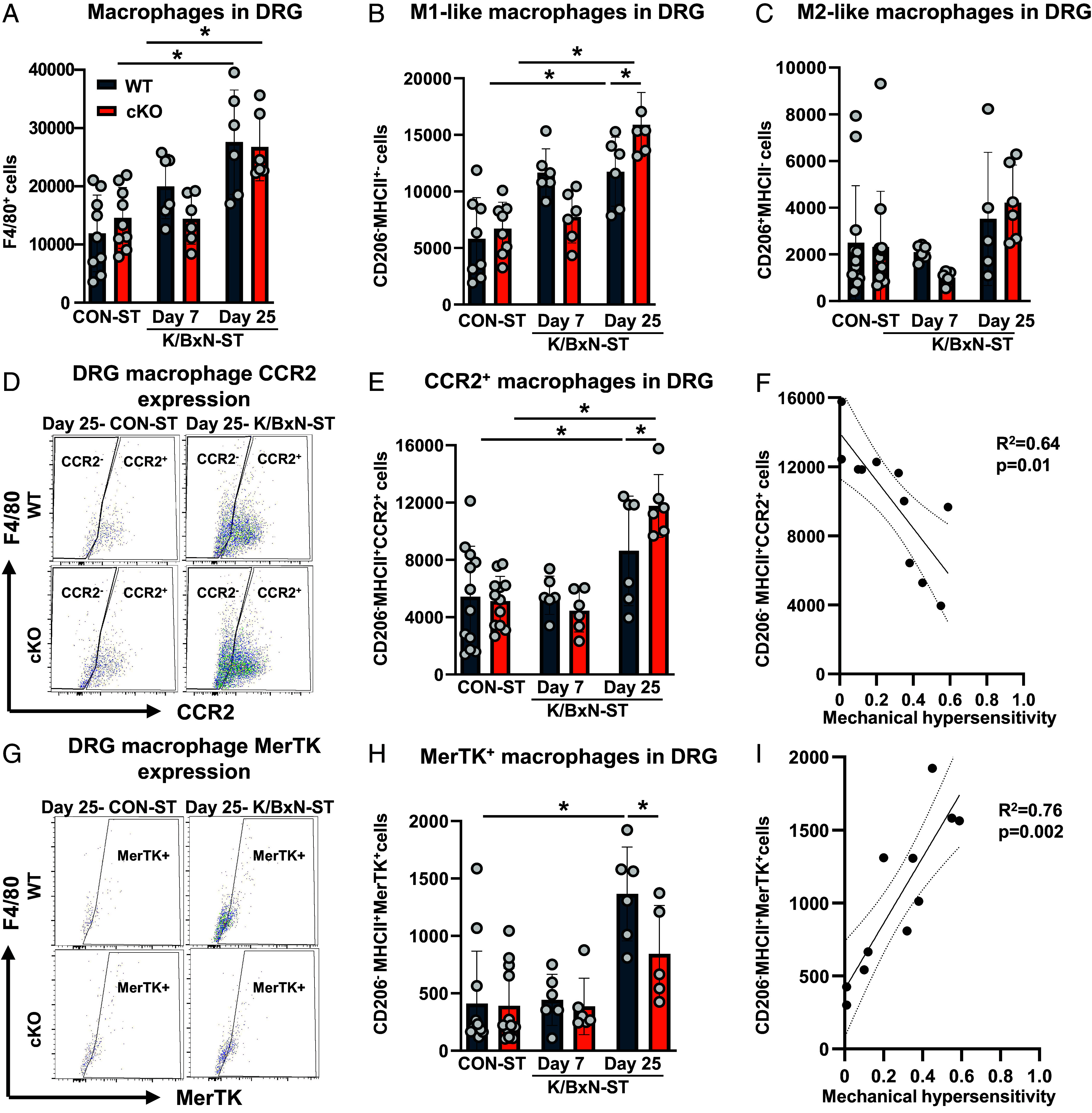
Modulation of macrophage subtypes in Cx3cr1^cre^Alox15^floxed^ DRG at day 25 after K/BxN-ST. (*A*) Quantification of L3–L5 DRG CD45^+^CD11b^+^F4/80^+^ macrophage, (*B*) F4/80^+^CD206^−^MHCII^+^ M1-like and (*C*) F4/80^+^CD206^+^MHCII^−^ M2-like macrophage absolute numbers. (*D*) Representative scatterplots of CCR2 expression in L3–L5 DRG CD206^−^MHCII^+^ macrophages. (*E*) Quantification of L3–L5 DRG CCR2^+^ macrophages absolute numbers. (*F*) Spearman’s correlation between absolute numbers of CD206^−^MHCII^+^CCR2^+^ macrophages and mechanical hypersensitivity (n = 11 biological replicates). (*G*) Representative scatterplots of MerTK expression in L3–L5 DRG CD206^−^MHCII^+^ macrophages. (*H*) Quantification of L3–L5 DRG CD206^−^MHCII^+^MertK^+^ macrophage absolute numbers. Data are mean ± SEM. n = 6 to 12 biological replicates. *P < 0.05, one-way ANOVA followed by Tukey’s multiple-comparison test. (*I*) Spearman’s correlation between absolute numbers of MerTK^+^ macrophages and mechanical hypersensitivity (n = 11 biological replicates).

Concerning macrophages, F4/80^+^ cells populated the DRG at day 25-K/BxN-ST in WT and cKO (Fig. 3*A*). Specifically, M1-like macrophage numbers (MHCII^+^ cells) were nearly double those in CON-ST DRG and more abundant in cKO than WT DRG (Day 25 only; Fig. 3*B*). In contrast, M2-like (MHCII^−^CD206^+^) macrophages were less abundant than M1-like cells in both cKO and WT DRG (Fig. 3*C*), indicating a proinflammatory status due to the M1/M2 ratio. Intriguingly, at day 25-KBxN-ST, we found that MHCII^+^CCR2^+^ macrophages were abundant in both WT and cKO and cell abundance correlated positively with allodynia (Fig. 3 *D*-*F*). Moreover, we identified a cluster of MHCII^+^ macrophages expressing Mer proto-oncogene tyrosine kinase (MerTK) in WT which was less abundant in cKO (Fig. 3 *G* and *H*) and negatively correlated with allodynia (Fig. 3*I*). The expression of TAM receptors in day 25-KBxN-ST DRG denotes presence of efferocytic macrophages. Plausibly, these cells aimed to clear apoptotic neutrophils which had infiltrated at earlier time points.

Overall, these data in WT and cKO show facilitation of monocyte and neutrophil infiltration and accumulation of M1-like macrophages. Furthermore, the Alox15 cKO results suggest that normally, in macrophages the enzymatic products of Alox15 exert an inhibitory control on leukocyte dynamics in DRG.

Then, to validate this hypothesis further and establish specificity of the DRG results, we determined leukocyte trafficking and phenotype in hind paws (flow cytometry gating strategy in *SI Appendix*, Fig. S2*C*) which are innervated by nociceptor peripheral terminals. Moreover, we assessed the extent of microgliosis in the dorsal horn of the spinal cord, which receives input from central terminals of paw nociceptors (flow cytometry gating strategy reported in *SI Appendix*, Fig. S2*D*).

We observed that like in WT, in cKO paws classical and nonclassical monocytes and neutrophils accumulated at peak swelling (day 7-K/BxN-ST), and cell numbers declined alongside resolution of swelling, though they were more abundant in cKO than in WT (day 25-K/BxN-ST) (*SI Appendix*, Fig. S3 *A*–*C*). In both WT and cKO paws, macrophage (F4/80^+^ cells) accumulation peaked at day 7- and diminished by day 25-K/BxN-ST (*SI Appendix*, Fig. S3*D*). Specifically, at day-7-K/BxN-ST, M1-like macrophage numbers were significantly higher than in CON-ST (*SI Appendix*, Fig. S3*E*). However, at day 25-K/BxN-ST, cKO and WT paw infiltrates contained ten times more M2 (MHCII^-^CD206^+^)- than M1 (MHCII^+^CD206^−^)-like macrophages and 25 to 30% of M2-like cells were MerTK^+^ resolving macrophages (*SI Appendix*, Fig. S3 *F* and *G*). M2-like macrophage prevalence over M1-like macrophages is a feature of the resolution phase of joint inflammation (18). Consistent with a proresolving environment in paws, in both WT and cKO, intraplantar injection of a MerTK activating antibody, activ-αMerTK (100 pmol/mouse) attenuated day 25-K/BxN-ST allodynia in ipsilateral, but not contralateral paws at 24 h after injection (*SI Appendix*, Fig. S3*H*). Thus, activation of proresolving macrophages in the paw environment attenuates persistent allodynia.

We further characterized the Alox15 cKO mice by measuring MaR1 content in paw homogenates and confirmed efficient silencing of *alox15* since in paw homogenates from CON-ST and day 25-KBxN-ST conditions, MaR1 content was approximately 200 and 100 pg/ml in WT and cKO, respectively (*SI Appendix*, Fig. S3*I*).

Thus, in WT and cKO, at peak allodynia and paw swelling, classical monocytes, neutrophils, and M1-like macrophages which infiltrate the inflamed paw may contribute to sensitization of nociceptive fibers by releasing pronociceptive factors. However, in the no longer-swollen paws, when mechanical hypersensitivity persists, M2-like macrophages represent 75% of the total myeloid cells and these proresolving cells are unlikely contributors to persistent nociceptive signaling. This contrasts with DRG leukocyte infiltrate that contains less M2-like than M1-like pronociceptive macrophages which represent 80% of total myeloid cells.

Such an outcome is in accordance with our and other’s evidence pointing to leukocyte trafficking in the DRG as more likely to play mechanistic roles in the periphery over leukocytes at the joint (9, 19) under persistent nociceptive conditions.

Concerning dorsal horn microglia, we found that in WT and cKO cell numbers (CX3CR1^+^P2Y12^+^ cells) were higher at both peak and persistent hypersensitivity time points compared to numbers in absence of allodynia in CON-ST dorsal horns (*SI Appendix*, Fig. S4*A*). Moreover, we confirmed efficient *alox15* silencing because in CON-ST and day 25-K/BxN ST, MaR1 contents were about 150 pg/mg in WT and 70 pg/mg in cKO dorsal horn homogenates (*SI Appendix*, Fig. S4*B*).

These results confirm the occurrence of a microglia response to K/BxN-induced inflammatory allodynia (20) which was comparable between WT and cKO. However, since MaR1 content in cKO dorsal horn was lower than in WT, it is possible that silencing Alox15 in microglia resulted in less production of antinociceptive MaR1 and this imbalance contributed to exacerbation of mechanical hypersensitivity in cKO. Since constitutive Cx3cr1-driven inactivation may not be microglia-specific (21), we will evaluate this hypothesis in follow-up studies using an inducible Cx3cr1-CreER that can be activated postnatally.

Overall, these data point to the DRG as the location along the pain pathway that displays the most significant difference between WT and cKO cell infiltrates. Thus, we focused on DRG as the primary site for immune cell trafficking and neuroimmune communication in relation to onset and persistence of pain-like behavior.

### Neutrophil Infiltration in DRG Is Driven by LTB_4_ Produced by Macrophages.

**Fig. 4.**
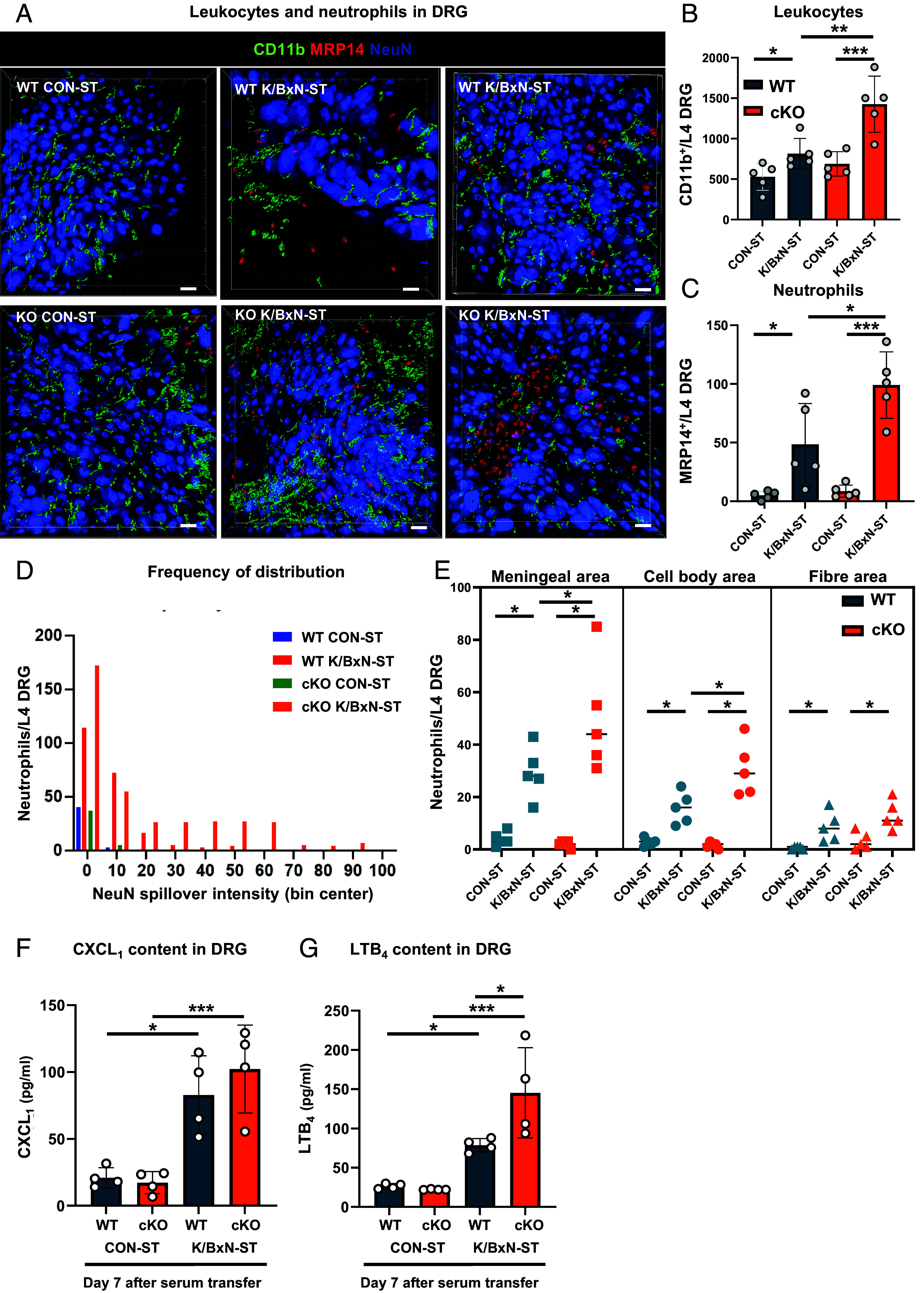
Neutrophils infiltration in Cx3cr1^cre^Alox15^floxed^ DRG at day 7 after K/BxN-ST. (*A*) Representative 3D confocal images of L4 DRG whole mounts to visualize neurons (NeuN), leukocytes (CD11b), and neutrophils (MRP14). (Scale bar, 30 μm.) (*B*) Quantification of leukocytes and (*C*) neutrophils. Data are mean ± SEM. n = 5 biological replicates. *P < 0.05, **P < 0.01, ***P < 0.001, one-way ANOVA followed by Tukey’s multiple-comparison test. (*D*) Frequency of distribution of NeuN intensity spilled over in neutrophils. (*E*) Quantification of neutrophils in meningeal area, cell body rich area and fiber rich area. Data are mean ± SEM. n = 5 biological replicates. In WT and cKO: *P < 0.05, one-way ANOVA followed by Tukey’s multiple-comparison test. (*F*) Quantification of CXCL_1_ and (*G*) LTB_4_ DRG levels. Data are mean ± SEM; n = 4 biological replicates. *P < 0.05, ***P < 0.001, one-way ANOVA followed by Bonferroni’s multiple-comparison test.

Having detected presence of neutrophils in cKO and to a lesser extent in WT DRG at peak inflammatory allodynia, we became intrigued by the possibility that these cells may infiltrate within the parenchyma. We were encouraged by recent evidence implicating neutrophil infiltration within DRG as mechanisms for widespread pain conditions (22). Using two-photon confocal microscopy, we found that, in accordance to flow cytometry analysis, in WT, leukocytes (CD11b^+^ cells) were more abundant at day 7-K/BxN-ST compared to CON-ST and accumulation was more pronounced in cKO DRG (Fig. 4 *A* and *B*). Neutrophils (MRP14^+^ cells) appeared interspersed among neuronal cell bodies (NeuN^+^ cells) and other leukocytes (CD11b^+^ cells) (Fig. 4*A*), but they were not in blood vessels (CD31^+^, *SI Appendix*, Fig. S5*A*). We quantified very few cells in CON-ST DRG of WT and cKO, few neutrophils in WT DRG at day-7-K/BxN-ST and double number of these cells in cKO DRG (Fig. 4*C*). A significant proportion (~35%) of neutrophils were located at the surface of DRG (bin center 10) (Fig. 4*D*). Some cells infiltrated WT parenchyma (bin 20 onward), and more neutrophils infiltrated cKO DRG (bins 60+) (Fig. 4*D*). Further distribution analysis showed that neutrophils were mostly located in meninges, over cell body and fiber-rich areas in WT and cKO DRG. However, several cells reached cell body areas in cKO and less so in WT (Fig. 4*E*). Next, we examined whether peripherally injected K/BxN IgG reached the DRG in view of recent evidence indicating that human IgG obtained from patients with fibromyalgia bind satellite cells in mouse DRG (23). However, we found that IgG immunostaining was visible in neither macrophages (F4/80^+^ cells) nor satellite cells (GFAP^+^ cells) in DRG tissue, suggesting that they had not penetrated the ganglia (*SI Appendix*, Fig. S5 *B* and *C*).

Thus, these data indicate that in concomitance to peak allodynia, neutrophils are recruited to DRG parenchyma and raise the possibility that neurons and/or macrophages attract neutrophils through chemokine and leukotriene production.

We already know that K/BxN-ST joint pathology depends on local neutrophil- and macrophage-mediated production of IL-1β (24, 25), and here we observed that this cytokine induced upregulation of neutrophil chemoattractant CXCL_1_ in cultured DRG neurons (*SI Appendix*, Fig. S6*A*). Consistent with this observation in vitro, in WT DRG, we detected CXCL_1_ upregulation at day 7-K/BxN-ST (Fig. 4*F*). However, in cKO DRG, CXCL_1_ levels were comparable to WT (Fig. 4*F*) ruling out that neuronal expression of CXCL_1_ accounts for larger neutrophil infiltration in cKO. Instead, neutrophil chemoattractant LTB_4_ levels were higher in cKO than WT DRG under K/BxN-ST conditions (Fig. 4*G*). In macrophages, LTB_4_ is produced from arachidonic acid (AA) by the enzyme 5-lipoxygenase (5-LOX) (26) (*SI Appendix*, Fig. S6*B*), which was expressed at a higher level in cKO BMDMs than WT (*SI Appendix*, Fig. S6*C*) and in cKO macrophages isolated from DRG in concomitance to Alox15 downregulation (*SI Appendix*, Fig. S6*D*). AA is produced from membrane phospholipids by phospholipase A2 (PLA2) (26), but we found that PLA2 content was comparable between cKO and WT macrophages (*SI Appendix*, Fig. S6 *E* and *F*), suggesting no alteration of AA biosynthesis in cKO macrophages.

Altogether these data suggest that DRG macrophages attract neutrophils via LTB_4_ production, and in cKO macrophages, this phenomenon is amplified because 5-LOX is up-regulated to balance against loss of Alox15. However, although macrophage production of LTB_4_ is a plausible mechanism to explain presence of neutrophils in cKO DRG parenchyma, we remained unclear on how macrophages were engaged by nociceptive neuron cell bodies. Since DRG neuron cell bodies can release EV (27) which comprise eicosanoids (28), we tested the hypothesis that EV shuttled AA to macrophages.

### Neuron-Derived EV are a Source of AA for 5-LOX-Mediated Conversion to LTB_4_ in Macrophages.

**Fig. 5.**
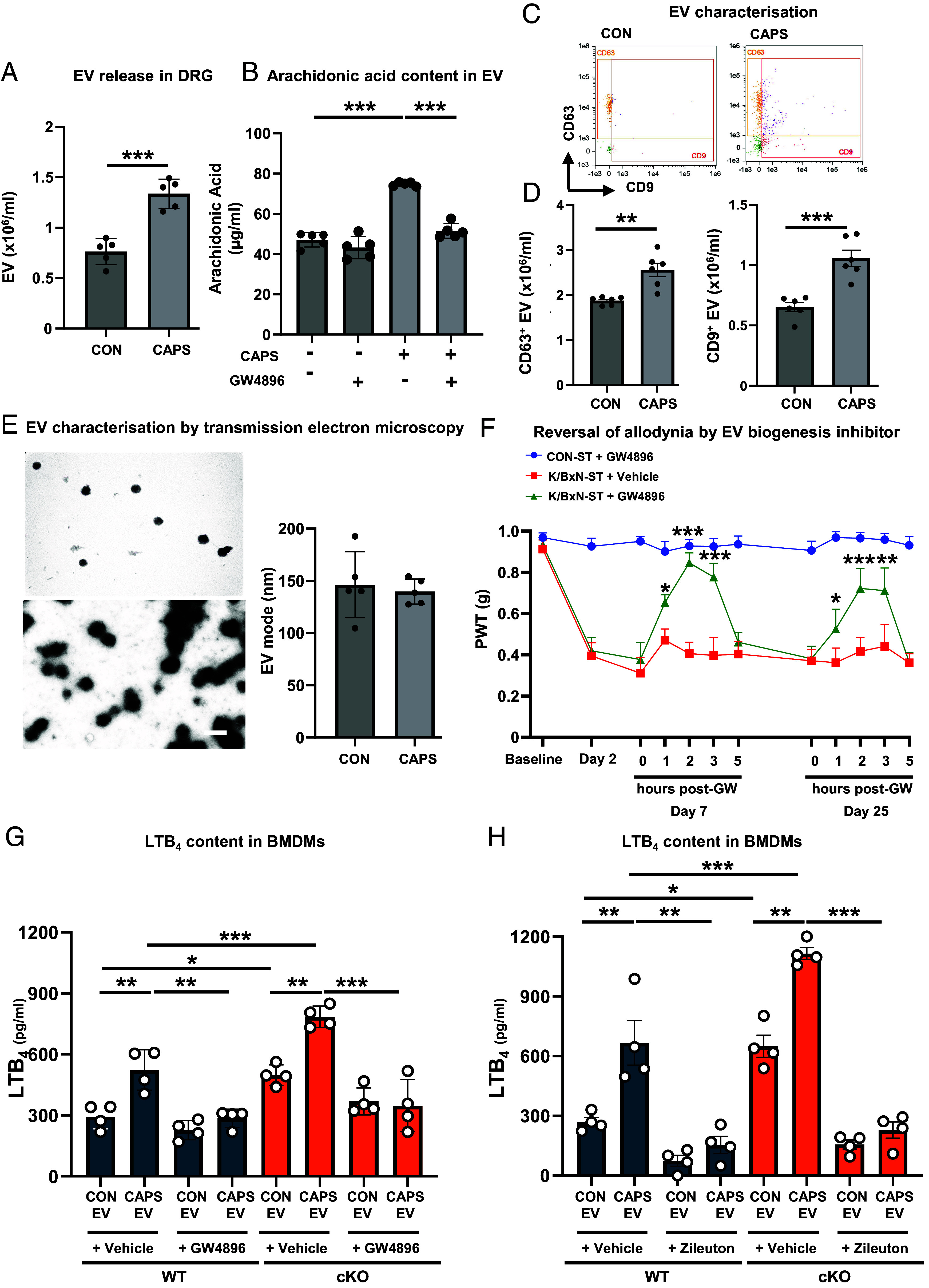
Sensory neuron extracellular vesicles (EV) provide the substrate for macrophages to produce LTB_4_ via 5-LOX activity. (*A*) Nanoparticle tracking analysis of EV isolated from culture media of DRG neurons incubated with vehicle (CON) or capsaicin (CAPS, 1 μM) for 3 h. Data are mean ± SEM; n = 5 biological replicates. ***P < 0.001, unpaired 2-tailed Student’s *t* test. (*B*) Quantification of arachidonic acid (AA) levels in EV isolated from culture media of DRG neurons incubated with either vehicle or capsaicin (CAPS) in presence and absence of GW4896 (5 μM). Data are mean ± SEM; n = 5 biological replicates.***P < 0.001, one-way ANOVA followed by Bonferroni’s multiple-comparison test. (*C*) ImageStream representative dot plots and (*D*) quantification of CD63^+^ and CD9^+^ EV isolated from culture media of DRG neurons. Data are mean ± SEM; n = 5 biological replicates. **P < 0.01; ***P < 0.001, unpaired 2-tailed Student’s *t* test. (*E*) Representative transmission electron microscopy images and quantification of mode of size of EV isolated from culture media of DRG neurons. (Scalebar, 300 nm.) (*F*) Intrathecal injection of GW4896 (GW; 100 pmol/mouse) reversed mechanical hypersensitivity in WT. Data are mean ± SEM, n = 8 mice per group (4 males and 4 females). *P < 0.05, **P < 0.01, ***P < 0.001, one-way ANOVA followed by Tukey’s multiple-comparison test. (*G*) Quantification of LTB_4_ levels in culture media of bone marrow–derived macrophages (BMDMs) treated with EV isolated from DRG neurons incubated with either vehicle (CON EV; number of EV 7.34 × 10^5^) or capsaicin (CAPS EV; number of EV 1.58 × 10^6^ EV) in presence and absence of GW4896. (*H*) Quantification of LTB_4_ levels in culture media of BMDMs treated with either CON EV or CAPS EV in presence and absence of 5-LOX selective inhibitor zileuton (100 μM). Data are mean ± SEM, n = 4 biological replicates. *P < 0.01, **P < 0.01, ***P < 0.001, one-way ANOVA followed by Bonferroni’s multiple-comparison test.

To assess whether neuroimmune communication via EV is operative in K/BxN ST DRG, first we confirmed that noxious-like activation of cultured DRG neurons by capsaicin-induced release of double the amount of EV than those accumulated in unstimulated neuron media (Fig. 5*A*). Then, as predicted, we observed that EV collected from DRG media contained approximately 100 to 250 µM AA (Fig. 5*B*). Furthermore, following capsaicin stimulation, more EV accumulation correlated with higher AA levels and changes in AA were genuinely associated to EV release, because addition of GW4869, an extracellular vesicle biogenesis inhibitor, normalized AA levels down to control values (Fig. 5*B*). Notably, we identified DRG EV as exosomes because i) they expressed tetraspanins CD9 and CD63 (Fig. 5 *C* and *D*), and ii) a large majority of vesicles (90%) displayed a diameter below 150 nm with a mode of ~90 nm (Fig. 5*E* and *SI Appendix*, Fig. S7 *A*–*D*). Next, to define possible contribution of EV release to allodynia, we delivered single intrathecal injections of GW4869 (100 pmol/mouse) at day 7- and day 25-KBxN-ST. We observed that GW4869 exerted antinociceptive effects at 1 and 2 h after injection that declined at 5 h and washed out by 24 h (Fig. 5*F*). Since GW4869 was antinociceptive at both day 7- and day 25-K/BxN-ST, these data imply that sustained release of EV in DRG contributes to onset and maintenance of mechanical hypersensitivity.

Then, using BMDMs, our first observation was that incubation with AA (30 µM to 1 mM) resulted in a concentration-dependent production of LTB_4_ (EC_50_ 195 and 123 µM in WT and cKO, respectively) which was more pronounced in cKO BMDMs (*SI Appendix*, Fig. S8). Our second observation was that exposure of BMDMs to neuron-derived EV resulted into LTB_4_ production by 5-LOX. Specifically, in WT BMDMs, we measured higher LTB_4_ levels following incubation with EV from capsaicin media than after either EV from vehicle media or capsaicin+GW4869 media (Fig. 5*G*). This outcome correlates with EV numbers which were the highest in capsaicin samples (1.58 * 10^6^ EV compared to 7.34 * 10^5^ in vehicle samples; Fig. 5*A*). In cKO BMDMs, LTB_4_ reached even higher levels than in WT (Fig. 5 *G* and *H*), an effect we ascribe to higher expression of 5-LOX. Indeed, in WT and cKO BMDMs, addition of zileuton, a selective 5-LOX inhibitor, inhibited the rise of LTB_4_ which was associated with incubation of EV from capsaicin media in absence of zileuton (Fig. 5*H*).

Altogether, these data delineate a pathway within DRG whereby following peripheral inflammation, nociceptive neuron cell bodies release EV-containing AA that are engulfed by macrophages. Through this mechanism, neurons transfer fatty acids to macrophages where 5-LOX metabolizes EV-derived AA into LTB_4_ promoting neutrophil infiltration in DRG parenchyma. The level of 5-LOX expression in macrophages is critical for this pathway to be functional as evident in cKO where neuron–macrophage–neutrophil communication is amplified because macrophages express high levels of 5-LOX due to absence of Alox15, and AA supplied by neurons is shunted to 5-LOX-mediated conversion into LTB_4_.

Since neutrophils are relatively short-lived cells, we were attracted by the possibility that programmed cell death of infiltrated neutrophils in DRG could promote macrophage efferocytosis and influence local macrophage phenotypes.

### Silencing Alox15 Is Associated with Impaired Efferocytosis and Proresolving Functions in Macrophages.

**Fig. 6.**
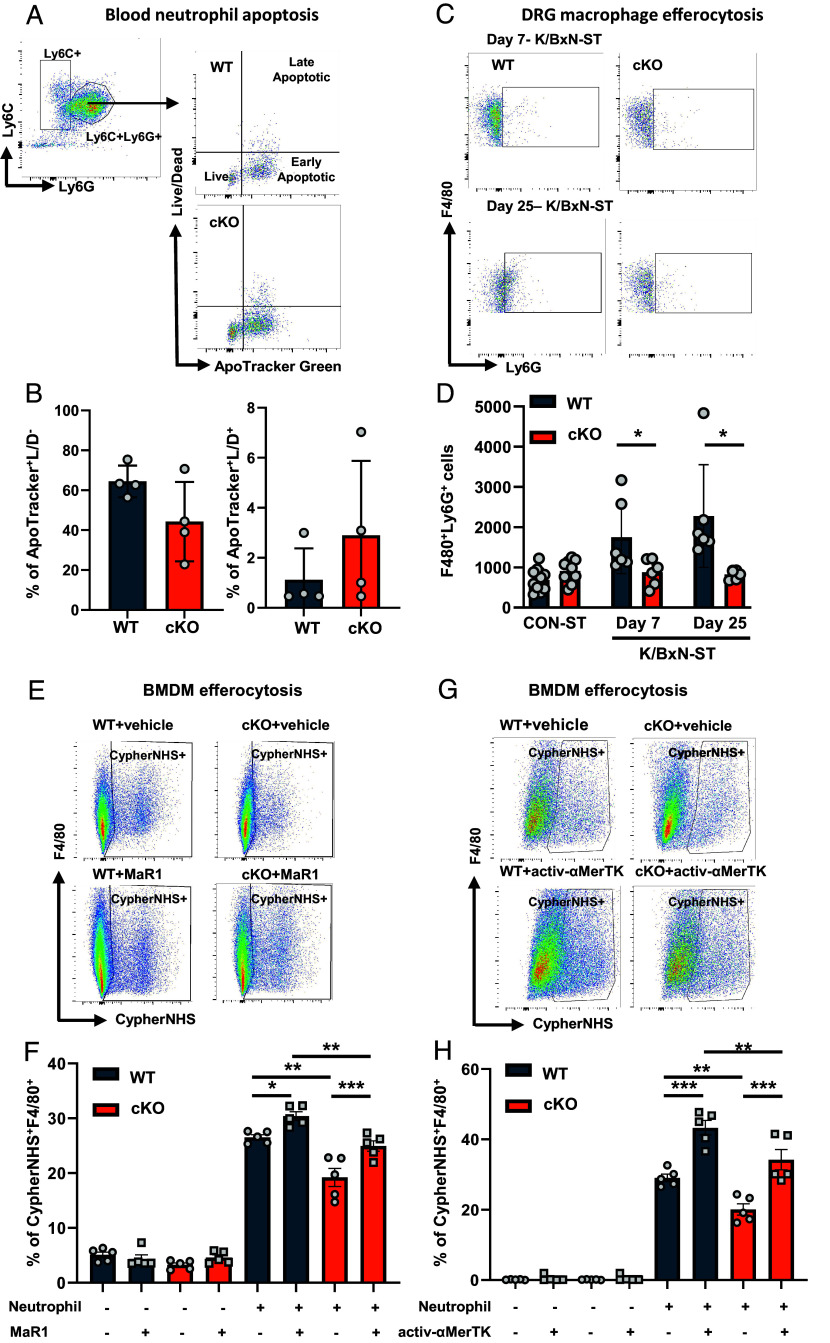
Impaired efferocytosis and proresolving functions of Cx3cr1^cre^Alox15^floxed^ macrophages. (*A* and *B*) Representative scatterplots of ApoTracker Green staining for apoptotic Ly6G^+^ neutrophils with quantification of early apoptotic (ApoTracker^+^/LD^−^) and late apoptotic (ApoTracker^+^/LD^+^) blood neutrophils populations. (*C* and *D*) Representative scatterplots and quantification of DRG Ly6G^+^F480^+^ macrophages. Data are mean ± SEM. n = 5 to 12 biological replicates. *P < 0.05, one-way ANOVA followed by Tukey’s multiple-comparison test. (*E* and *F*) Representative scatterplots and quantification of F4/80^+^/CypherNHS^+^ BMDMs in presence and absence of Maresin 1 (MaR1; 100 nM) and (*G* and *H*) activating MerTK antibody (activ-αMerTK; 5 nM). Data are mean ± SEM. n = 5 biological replicates. *P < 0.05; **P < 0.01; ***P < 0.001, one-way ANOVA followed by Bonferroni’s multiple-comparison test.

Concerning neutrophil apoptosis, we observed that early (Apotracker^+^/L/D^−^ cells) and late (Apotracker^+^/L/D^+^ cells) apoptosis was comparable in neutrophils obtained from blood of WT and cKO mice (Fig. 6 *A* and *B*). We know that apoptotic neutrophils are rapidly engulfed by macrophages through the process of efferocytosis (29) and here we found WT macrophages containing apoptotic neutrophils in DRG at day 7- and day 25-KBxN-ST (*SI Appendix*, Fig. S9). DRG cKO macrophages (F4/80^+^ cells) engulfed less apoptotic (L/D^+^Ly6G^+^) neutrophils than WT regardless of whether they were isolated from day 7- or day 25-KBxN-ST DRG (Fig. 6 *C* and *D*). These data suggest impaired efferocytosis by cKO macrophages which we explored further by testing molecules that promote macrophage efferocytosis of apoptotic neutrophils such as MaR1 and activ-αMerTK antibody (30).

In line with cKO DRG macrophages, we observed that cKO BMDMs (F4/80^+^ cells) performed less efferocytosis of apoptotic neutrophils (F4/80^+^CypherNHS^+^ cells) when compared to WT BMDMs (Fig. 6 *E* and *G*). MaR1 (100 nM) addition to BMDMs enhanced efferocytosis in WT and partially rescued efferocytosis in cKO (Fig. 6 *E* and *F*). Similarly to MaR-1, the addition of activ-αMerTK antibody to BMDMs also increased efferocytosis in WT and moderately restored efferocytosis in cKO when measured by both flow cytometry and immunohistochemistry (Fig. 6 *G* and *H* and *SI Appendix*, Fig. S10 *A*–*D*). The effects of activ-αMerTK antibody and MaR1 were not secondary to modulation of efferocytic receptors, as cell-surface levels of MerTK were comparable between WT and cKO BMDMs (*SI Appendix*, Fig. S10 *E*–*H*).

Collectively, these data indicate that Alox15 silencing compromises macrophage proresolving functions (efferocytosis) that is induced by neutrophil apoptosis. Thus, in our final step, we boosted proresolving DRG macrophages by intrathecal administration of MerTK activating antibody.

### Intrathecal Delivery of MerTK Activating Antibody Inhibits Inflammatory Arthritis Allodynia via Polarization of DRG Macrophages Toward Proresolving Phenotype.

To assess whether activation of MerTK in macrophages inhibited nociceptive processing within the DRG, we monitored day 25-K/BxN-ST mechanical hypersensitivity after intrathecal administration of activ-αMerTK (MerTK activating antibody) and examined DRG macrophage phenotype by flow cytometry analysis immediately after behavior. Furthermore, considering that MerTK activation facilitates 5-LOX mediated release of proresolving and antinociceptive lipid mediator lipoxin A4 (LXA_4_) (31) (*SI Appendix*, Fig. S11*A*), we also measured LXA_4_ DRG levels by ELISA.

**Fig. 7.**
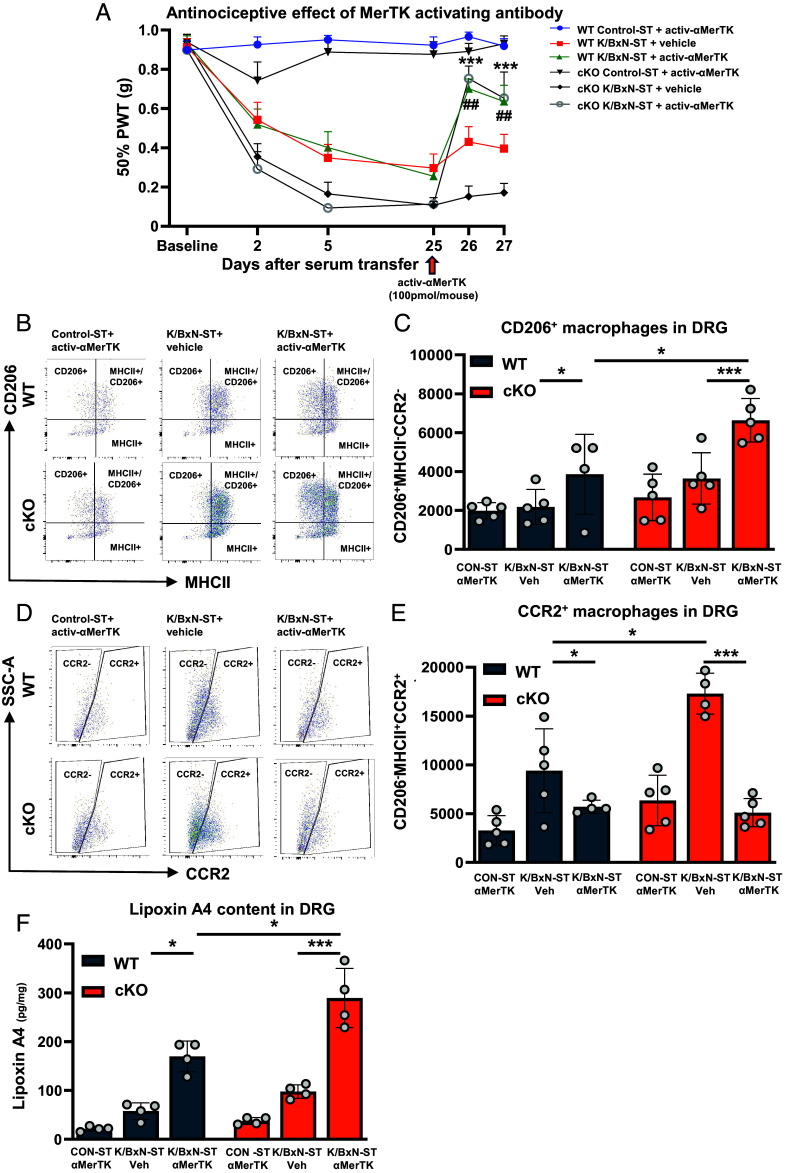
Antinociceptive effect of MerTK activating antibody via polarization of DRG macrophages toward proresolving phenotype. (*A*) Intrathecal injection of activating MerTK antibody at day 25-K/BxN-ST (activ-αMerTK; 100 pmol/mouse) reversed mechanical hypersensitivity in WT and cKO. Data are mean ± SEM, n = 8 mice per group (4 males and 4 females). In cKO K/BxN-ST same-day: ***P < 0.001 vehicle vs activ-αMerTK; In WT K/BxN-ST same-day ^##^*P* < 0.01 vehicle vs activ-αMerTK. (*B*) Representative scatterplots and (*C*) quantification of L3–L5 DRG CD206^+^MHCII^-^CCR2^−^ macrophage absolute numbers, at day 27, i.e., 48 h after i.t. delivery of activ-αMerTK (αMerTK). (*D*) Representative scatterplots and (*E*) quantification of CD206^−^MHCII^+^CCR2^+^ macrophage absolute numbers in L3–L5 DRG at day 27, i.e., 48 h after i.t. delivery of activ-αMerTK (αMerTK). (*F*) Quantification of DRG Lipoxin A4 (LXA_4_) levels at day 27, i.e., 48 h after i.t. delivery of activ-αMerTK (αMerTK). Data are mean ± SEM. n = 5 biological replicates. *P < 0.05, ***P < 0.001 one-way ANOVA followed by Tukey’s multiple-comparison test.

We observed attenuation of day 25-K/BxN-ST allodynia at 24 and 48 h after single injection of activ-αMerTK (100 pmol/mouse) in WT and cKO compared to injection of vehicle (Fig. 7*A*).

Analysis of DRG macrophage clusters in KBxN-ST mice treated with activ-αMerTK antibody compared to vehicle-treated mice revealed the following scenario: Numbers of MHCII^+^MerTK^+^CD206^−^ macrophages were unaltered (*SI Appendix*, Fig. S11*B*), perivascular resident MHCII^-^CD163^+^ macrophages [identified as in (32)] were higher in WT and cKO DRG (*SI Appendix*, Fig. S11*C*), MHCII^-^CD206^+^CCR2^−^ (M2-like) macrophages were higher in WT and even more abundant in cKO (Fig. 7 *B* and *C*), MHCII^+^CCR2^+^CD206^−^ (M1-like) macrophages were decreased in WT and even more in cKO DRG (Fig. 7 *D* and *E*). Moreover, in DRG of WT K/BxN-ST, LXA_4_ levels were higher following activ-αMerTK antibody compared to vehicle treatment (Fig. 7*F*). In DRG of cKO KBxN-ST, LXA_4_ reached even higher levels than in WT following activ-αMerTK treatment (Fig. 7*F*), an effect we ascribe to higher expression of 5-LOX. These data show that activ-αMerTK antibody administration resulted in polarization of DRG macrophages toward proresolving (M2-like) phenotype which release antinociceptive lipid mediator LXA_4_. Furthermore, the higher levels of LXA_4_ in cKO than in WT DRG correlate with activ-αMerTK antibody 90% and 50% reversal of allodynia in cKO and WT, respectively.

## Discussion

This study delineates a neuroimmune pathway within the DRG that mediates mechanisms underlying persistent inflammatory arthritis pain and offers therapeutic targets for such a condition. We have identified specific neuroimmune interactions and delineated a neuron–macrophage–neutrophil bidirectional circuit which maintains nociceptive signaling responsible for persistent allodynia even when inflammation resolves in the arthritis joint.

Tissue-specific cellular and humoral processes occur in arthritis, and our focus here is at the DRG level. In our in vitro settings, noxious-like activation of DRG neuron cell bodies results in release of EV that provide macrophages with AA substrate for 5-LOX to produce neutrophil chemoattractant LTB_4_. EV are characterized by a double layer of lipids, with asymmetric distribution of phospholipids so that phosphatidylserine is exposed to the outside and serves as an “eat-me” signal for macrophages (33). AA is contained in EV and it is the substrate source for the synthesis of LTB_4_ by macrophages in DRG. Indeed, our ex vivo evidence indicates that LTB_4_ is produced in DRG alongside neutrophil infiltration and in concomitance to paw swelling and allodynia. EV release is causal to inflammatory pain mechanisms because intrathecal delivery of GW4869, EV biogenesis inhibitor (34), reverses both peak and persistent mechanical hypersensitivity. Despite our main interest being in making a comparison between WT and cKO, we have pondered whether K/BxN and control sera contain EV and AA which may access the DRG. However, we think it is highly unlikely that the serum itself causes the changes we observe based on the following two observations: i) Control serum failed to affect nociceptive thresholds and ii) K/BxN and control IgG failed to penetrate DRG parenchyma in contrast with human IgG obtained from patients with fibromyalgia that bind satellite cells in DRG (23). Alongside EV release by neurons, macrophage expression of 5-LOX is rather critical because the higher the levels of such enzyme in absence of Alox15 the more LTB_4_ is formed, and more neutrophils infiltrate the DRG parenchyma. Neutrophil infiltration occurs within 7 d from induction of ankle joint inflammation and allodynia. Then, spontaneous neutrophil apoptosis within the DRG likely impacts macrophage phenotype by promoting efferocytosis (35). We know that the process of efferocytosis is a potent polarizing event for macrophages, which release proresolving molecules that modify the surrounding environment (17). Indeed, we found clusters of efferocytic macrophages in DRG at around two weeks from neutrophil infiltration in concomitance to persistent inflammatory allodynia. Efferocytic macrophages perform proresolving functions including production of proresolving lipid mediators such as MaR1 produced by Alox15 and LXA_4_ produced by 5-LOX (17, 31, 36). In our settings, Alox15 silencing in macrophages results in low DRG levels of antinociceptive MaR1 and high levels of pronociceptive PGE_2_ that correlate with exacerbation of inflammatory allodynia. Notably, in cKO, 5-LOX upregulation that balances out Alox15 loss, results in a modest increase of LXA_4_ which is insufficient to overcome the effects of pronociceptive factors such as PGE_2_. However, LXA_4_ DRG levels increase significantly following promotion of efferocytosis by activ-αMerTK antibody which impacts the local DRG microenvironment with higher proportion of M2-like macrophages and lower numbers of M1-like macrophages. This process explains accumulation of more CD206^+^ macrophages in DRG after treatment with MerTK activator, even if proportion of MerTK^+^ macrophage before treatment was relatively low. Such a modulation of macrophage clusters, including those positive for MerTK, is plausibly mediated by soluble factors released by efferocytotic macrophages, as reported in other settings (17). One of such mediators is LXA_4_ which is synthesized downstream 5-LOX (7), and consistently with upregulation of 5-LOX in cKO macrophages, it is produced at higher levels in cKO than WT DRG in concomitance with accumulation of more M2-like macrophages after MerTK activation.

Neutrophil presence in the DRG parenchyma is an interesting observation as it indicates the occurrence of a unique environment in the DRG in response to neuronal activity in the context of inflammatory arthritis pain. Indeed, we suggest that neurons are a likely source of CXCL_1_ in day 7-KBxN-ST DRG in both WT and cKO and this chemokine is a potent chemoattractant for neutrophils that also respond to LTB_4_ produced by macrophages (37). We envision neutrophils as normally residing within membranes around the DRG and attracted into the parenchyma in WT and to a greater extent in cKO where LTB_4_ production is higher. In inflammatory pain, this phenomenon appears clearer than in DRG following peripheral nerve injury where it remains a controversial issue as neutrophils were reported to either populate the meninges without invading the parenchyma (38) or invade the parenchyma (39). Besides neutrophil chemoattractant functions, CXCL_1_ and LTB_4_ are likely to exert pronociceptive action via activation of neuronal CXCR2 and BLT1 receptors, respectively (40, 41). In a similar fashion, proinflammatory lipid mediator such as macrophage derived PGE_2_ would activate EP2 receptor in neurons and facilitate nociceptive signaling. Instead, MaR1 and LXA_4_ would inhibit nociceptor activity via leucine-rich repeat-containing G-protein-coupled receptor 6 (LGR6) and formyl peptide receptor 2 (ALX/FPR2), respectively (30, 42).

In summary, we report that under inflammatory pain conditions, DRG neuron cell bodies release EV that are transferred to macrophages where AA is converted by 5-LOX into LTB_4_ which attracts neutrophils in DRG parenchyma (*SI Appendix*, Fig. S12). This pathway is validated in cKO macrophages that produce more LTB_4_ and attract more neutrophils in DRG. Subsequent neutrophils apoptosis promotes macrophage efferocytosis, and DRG macrophages acquire proresolving phenotype associated with release of MaR1. Such an efferocytic function is significantly impaired in cKO macrophages that produce lower amount of MaR1. However, in both WT and cKO conditions, formation of antinociceptive MaR1 is not sufficient to counteract production and function of pronociceptive lipid mediator PGE_2_ which maintains nociceptive processing at the DRG level. Nevertheless, in both WT and cKO, promotion of macrophage efferocytosis by the MerTK activating antibody, results in an antinociceptive effect which lasts for at least 48 h. This type of behavior is associated with DRG macrophage polarization toward M2-like phenotype and production of antinociceptive lipid LXA_4_ which redresses the imbalance of proresolving lipid mediator synthesis by macrophages. These proof-of-concept data suggest that DRG MerTK+ macrophages are a possible target for pain in RA in a similar fashion to synovial MerTK+ macrophages which have been proposed as targets for remission of RA (43). Current therapies for pain in RA are reliant on nonsteroidal anti-inflammatory drugs, which target lipid biosynthesis by inhibiting COX-1 and COX-2. Our and other’s works open avenues of exploration for other lipid mechanisms such as those responsible for production of proresolving lipid mediators that result imbalanced in persistent inflammatory arthritis pain.

The outcome of this study reinforces the concept that pain results from cooperation of immune and nervous systems (44, 45) and provides modalities of neuroimmune interactions in the periphery that are specific to persistent inflammatory arthritis pain. Thus, we suggest that redressing the imbalance of proresolving lipid mediator synthesis by macrophages, would modulate nociceptive neuron activity and offer an alternative approach for therapeutic analgesia. We propose that boosting proresolving macrophages in the periphery, including the DRG and the joint is an innovative approach to resolve persistent arthritis pain.

## Materials and Methods

Studies were conducted in male and female C57BL/6 mice. Alox15‐deficient (cKO) mice were generated by crossing loxP‐flanked Alox 15 mice with mice expressing Cre‐recombinase under the *Cx3cr1* gene promoter. Experiments were performed under a United Kingdom’s Home Office License P998AB295.

Inflammatory arthritis was induced by injecting K/BxN serum (50 μl i.p.) on day 0 and 2. Clinical scores, ankle thickness, and hind paw mechanical thresholds were acquired at several time-points. GW4896 (100 pmol/5 μl) was injected intrathecal and activ-αMertK antibody (100 pmol/5 μl) intrathecal and intraplantar.

**Flow cytometry** analyses of paw cells required staining with antibodies listed in *SI Appendix*, Table S1. Single-cell suspensions from DRG and microglia were characterized with antibody mix as in *SI Appendix*, Table S2.

**Whole mount ex vivo multiphoton microscopy** was performed in fixed and permeabilized DRG stained for MRP14, CD11b, CD31, and NeuN. Images produced by confocal microscopy were analyzed with IMARIS software.

**Immunohistochemistry** of DRG sections was performed with antibodies against F4/80, MRP14, and GFAP as in Supporting information file. Apoptotic cells detected with TUNEL assay.

**RT-qPCR** was performed using Mm_Alox15_1_SG and Mm_Pla2g4a_1_SG primers (Qiagen). Gene expression levels were normalized to 18S housekeeping gene.

Apoptotic neutrophils were labeled with CypHer5E. Macrophage efferocytosis was measured in BMDMs treated with MaR1 (100 nM) or activ-αMerTK antibody (5 nM), prior to addition of apoptotic neutrophils. Ly6G neutrophil expression was quantified in F4/80 positive macrophages by flow cytometry in single-cell suspensions from DRG. Engulfed apoptotic neutrophils were visualized by immunohistochemistry.

EV were collected from cultured DRG neuron media and analyzed using Nanosight NS300 (Malvern Instruments) ImageStream™ and Transmission Electron microscopy using JEOL 1400+ TEM equipped with an AMT XR16 CCD camera for images.

**ELISA** was used to quantify MaR1 in DRG, paw, and dorsal horn homogenates, LTB_4_, MaR1, PGE_2_, LXA_4_, and CXCL_1_ in DRG extracts, LTB_4_ in BMDMs supernatants following removal of EV, and AA in sonicated EV.

Extensive *Material and Methods* are available in *SI Appendix*.

## Supplementary Material

Appendix 01 (PDF)

## Data Availability

All study data are included in the article and/or *SI Appendix*.
