# Supplementary material for: Activation of proresolving macrophages in dorsal root ganglia attenuates persistent arthritis pain
Source: Proc Natl Acad Sci U S A. 2025 Mar 10;122(11):e2416343122. doi: 10.1073/pnas.2416343122 (PMC11929478; doi:10.1073/pnas.2416343122)
Supplement: Supplementary file 1 — Appendix 01 (PDF) [file pnas.2416343122.sapp.pdf]

PNAS

Supporting information for

Activation of pro-resolving macrophages in dorsal root ganglia attenuates persistent arthritis pain

Silvia Oggero[1], Mathieu-Benoit Voisin[2], Francesca Picco[1], Miguel Á. Huerta[1,5], Chiara Cecconello[2], Thomas Burgoyne[3,4], Mauro Perretti[2], Marzia Malcangio[1*]

1. Sensory, Pain and Regeneration Centre, Kings College London, Guys' Campus, London Bridge, London SE1 1UL, United Kingdom

2. William Harvey Research Institute, Faculty of Medicine and Dentistry, Queen Mary University of London, Charterhouse Square, London EC1M 6BQ, United Kingdom

3. UCL Institute of Ophthalmology, University College London, London EC1V 9EL, UK

4. Royal Brompton Hospital, Guy's and St Thomas' NHS Foundation Trust, London SW3

6NP, United Kingdom

5. Department of Pharmacology, University of Granada, 18016 Granada, Spain.

*Marzia Malcangio. Wolfson SPaRC, King's College London, Guy's Campus, London Bridge, London SE1 1UL;

Email: marzia.malcangio@kcl.ac.uk; ORCID ID: 0000-0003-4296-5727PNAS

This PDF file includes:

Supporting materials and methods
Figures S1 to S12
Tables S1 and S2
SI references

Material and methods

Animals
All studies were conducted in C57BL/6 black male and female mice. *Cx3cr1*:cre-driven Alox15-deficient (Alox15 cKO) mice were generated as previously described (1) by crossing loxP-flanked (floxed) Alox 15 mice (B6.Cg-Alox15$^{tm1.1Nadl/J}$; strain #03 1835; kindly donated by Jesmond Dalli) with mice expressing Cre-recombinase from the *Cx3cr1* gene promoter (B6J.B6N(Cg)-Cx3cr1$^{tm1.1(cre)Jung/J}$; strain #02 5524; kindly donated by Jesmond Dalli). Experiments were performed under a United Kingdom's Home Office Licence (MM Project Licence P998AB295) and procedures performed while adhering to the UK's Guidance on the Operation of Animals, Scientific Procedures Act (1986), and Laboratory Animal Science Association Guidelines' Guiding Principles on Good Practice for Animal Welfare and Ethical Review Bodies. Mice were housed on a 12-hour light/dark cycle with *ad libitum* standard laboratory diet and water. Adult 8- to 12-week-old cKO mice and their control littermates were randomly assigned to groups and each group contained the same number of age-matched mice of both sexes.

Induction of K/BxN serum transfer inflammatory arthritis
Inflammatory arthritis was induced using previously described protocols (2, 3). On days 0 and 2, mice received intraperitoneal injections of K/BxN serum (50 µl); control mice received serum from KRN/C57 mice (non-arthritic). Clinical signs of arthritis were assessed using a 12-point scoring system, with each limb scored separately; 0 to 3 points per limb with the following criteria: 0, no sign of redness/swelling; 1, redness/swelling observed in either ankle/wrist, pad, or any of the digits; 2, redness/swelling in 2 regions; and 3, redness/swelling seen in all limb sections. Scores for all four limbs were combined in a total score with a maximum of 12 per animal. Ankle thickness was measured using a digital calliper, and joint measurements were taken before the first serum injection and repeated daily after serum injection.

Intrathecal injections
Intrathecal injections were performed under light isoflurane anaesthesia into the lumbar region of the spinal cord (between L4 and L5 vertebrae) using a 30-gauge needle. GW4896 (D1692, Merck) was injected at 100 pmol/5ul/mouse (0.001% DMSO) and MertK activating antibody (AF591, R&D) at 100 pmol/5ul/mouse dissolved in phosphate-buffered saline (PBS).

Intraplantar injections
Intraplantar injections were performed under light isoflurane anaesthesia into the plantar surface of the hind paw using a 30-gauge needle. MertK activating antibody (AF591, R&D) at 100 pmol/10ul/mouse dissolved in phosphate-buffered saline (PBS).

Behavioural testing
To assess hind paw mechanical thresholds, calibrated von Frey monofilaments (ranging from 0.008 to 1.0 g) were applied to the plantar surface. The testing began after 30 min of habituation during the light cycle, with a 0.07-g filament applied until the paw withdrew (unrelated to movement or grooming). Filaments were alternately applied to the left and right hind paws. The 50% withdrawal thresholds were determined using the up-and-down method, adjusting the force based on positive or negative responses to the 0.07-g filament (4). The experimenter was blind to treatments and mouse genotypes.

Flow cytometry of paw tissue
Leukocytes were isolated following tissue digestion, which involved cutting paws 3 mm above heels, removing skins, and disarticulating fingers using blunt forceps. Tissues were digested twice in 15 ml of buffer (containing Collagenase D [Roche; 0.5 µg/ml] and DNAse [Sigma-Aldrich; 40 µg/ml]) in serum-free RPMI with gentle agitation for 30 min at 37 °C. Cells were filtered through a 70 µm strainer and kept on ice in 2 ml of ice-cold fetal bovine serum solution (FBS qualified HI, Gibco). After centrifugation for 10 min at 400xg, cells were resuspended in PBS (Sigma) for staining. Cells were first incubated with anti-CD16/CD32 (101302, Biolegend 1:1000, 15 min, 4°C) antibody to block nonspecific Fc receptor binding and then stained with Fixable Viability Dye eFluor™ 780 (65-0865-18,Thermofisher, 1:1000) to identify live cells. Distinct leukocyte subtypes were identified using antibodies listed in Table S1. Samples were run using LSRFortessa cell analyser (BD Bioscience) and analysed using FlowJo software (v10.1; Tree Start, Inc). All gating strategies were generated using Fluorescent Minus One (FMO) controls.

Flow cytometry of dorsal root ganglia
To obtain the dorsal root ganglia (DRG), mice were irreversibly anesthetized by intraperitoneal injection of pentobarbital (40 mg/kg; Pentoject) and perfused with ice-cold PBS. L3-5 DRG were rapidly dissected bilaterally and placed into ice-cold HBSS (Gibco). Single-cell suspensions were obtained after enzymatic digestion using 3 mg/ml Dispase (Roche), 0.125% collagenase (Sigma-Aldrich), and 200 U/ml DNase I (Sigma-Aldrich) in F-12 medium (Gibco) for 45 min at 37°C, followed by trituration and centrifugation at 300xg for 5 min. Single cells were resuspended in PBS and stained for Zombie NIR™

Fixable Viability kit (BioLegend) for 15 min at room temperature, followed by staining with directly conjugated antibody mix for 30 min using antibodies listed in Table S2. Cells were then washed, centrifuged (1 min, 300xg), and resuspended in flow buffer containing 15 µl of CountBright™ Absolute Counting Beads (C36950, Thermofisher scientific) before being analysed. Samples were run using LSRFortessa cell analyser (BD Bioscience) and analysed using FlowJo software (v10.1, BD Bioscience). All gating strategies were generated by using Fluorescent Minus One (FMO) controls. For another set of experiment DRG immune cells were first stained for CD45.1 BV786 (103139, 5 µg/ml, clone 30-F1, BioLegend), F4/80 PE (12-4801-82, 0.25 µg/ml, clone BM8, ThermoFisher scientific), CD11b BV421 (101236, 2 µg/ml, clone M1/70, BioLegend), and then permeabilized and fixed using the Foxp3 / Transcription Factor Staining Buffer Set (00-5523-00, eBioscience) according to manufacturer's instructions. Permeabilized cells were stained with fluorophore-conjugated antibodies against ALOX15 AF647 (BS-3874R, Bioss) and 5-LOX AF488 (BS-352R, Bioss) or Ly6G AF700 (127622, 1 µg/ml, clone 1A8, Biolegend) for 30 min at 4 °C.

Flow cytometry of dorsal horn microglia

Mice were deeply anesthetized by intraperitoneal injection of pentobarbital (40 mg/kg; Pentoject) and perfused with ice-cold PBS. Dorsal horns of the spinal cord were quickly dissected after removal of the meninges and placed in PBS on ice. Microglia isolation was carried out following a published protocol (5, 6). Briefly, tissue was mechanically homogenized using loose and tight pestles in ice-cold PBS and resulting cell suspension was filtered through a pre-wet 70-µm cell strainer. To remove myelin, 100 ul of Myelin Removal Beads II (130-096-433, Miltenyi Biotec) were added per dorsal horn and processed using pre-wet LS columns (130-042-401, Miltenyi Biotec) on a MACS magnet stand. Finally, cells from the flow-through were collected, washed, and prepared for standard FACS staining. Single cells were resuspended in PBS and stained for Zombie NIR™ Fixable Viability kit (BioLegend) for 15 min at room temperature, followed by staining with directly conjugated antibody mix as stated in Table S2 for 30 min.. Cells were then washed, centrifuged (1 min at 300xg), and resuspended in flow buffer containing 15 µl of CountBright™ Absolute Counting Beads (C36950, Thermofisher scientific) before being analysed. Samples were run using LSRFortessa cell analyser (BD Bioscience) and analysed using FlowJo software (v10.1, BD Bioscience). All gating strategies were generated by using Fluorescent Minus One (FMO) controls.

Whole mount *ex vivo* multiphoton microscopy

Mice were deeply anesthetized (pentobarbital at 40 mg/kg; Pentoject) and perfused with ice-cold PBS. DRG were immediately harvested and incubated for 16 h with 4% PFA at 4˚C followed by incubation with perm block for 4 h (20% serum, 1% Triton X- in PBS). DRGs were then incubated for 48 h at 4˚C with primary antibodies against MRP14 (ab105472, 1:200; abcam), CD31 AF647 (102515, 1:200; BioLegend), and NeuN (ab190565, 1:500; Abcam), CD11b AF594 (ab312914, 1:100, abcam). Anti-MRP14 was conjugated with AF555, carried out using labelling kit (A20187, Thermo Fisher Scientific) according to the manufacturer's instructions. Samples were prepared for imaging by PBS immersion and covered with a coverslip. DRGs were imaged with a Leica SP8 DIVE multiphoton confocal microscope (Leica Microsystems) equipped with a 25× 1.0 NA WI IR objective lens and a pulsed infrared laser. Most experiments were performed at 795 nm and 1,045 nm (MP laser) excitation with an intensity between 20.1% and 25%, respectively. Images were acquired with a 0.50-µm z step size with approximate z depth of 150 µm. Predefined settings for laser power and detector gain (speed 8,000, pixel size 346.32 nm^2) were used for all experiments. Three-dimensional images were then analysed offline using IMARIS software (Bitplane, Switzerland), and the number of neutrophils, $CX3CR1^+$ and $CD11b^+$ per field of view was quantified by creating isosurfaces on their respectively channels.

Immunohistochemistry of dorsal root ganglia

Transverse DRG sections (10 µm) were then cut on a cryostat (Bright instruments) and mounted onto Superfrost Plus microscope slides (ThermoFisher scientific). Sections were permeabilised with PBS with 0.1 % Triton-X-100 (PBS-T; Sigma-Aldrich) for 15 min, blocked with 3 % BSA (Sigma-Aldrich) for 1 h and incubated overnight with antibodies against F4/80 (ab6640, 1:100; Abcam), MRP14 (ab105472, 1:200; Abcam), mouse IgG (ab175699, 1:100; Abcam), GFAP (MAB360, 1;1000;Millipore) followed by appropriate secondary antibodies (1 µg/ml, Invitrogen) for 1 h. Slides are then washed 3 times and mounted in DAPI containing mounting solution (Invitrogen). For apoptosis experiment, fixed DRG were stained for apoptotic cells using Click-IT Plus TUNEL assay (C10619; Invitrogen) according to manufacturer instructions. Images for immunofluorescence analysis were captured using a Zeiss LSM400 fluorescence microscope and analysed using ImageJ software (1.50i, Wayne Rasband, National Institutes of Health, USA).

Primary cultures of bone marrow derived macrophages

BMDMs from WT and cKO were collected and generated as previously described (7, 8). Briefly, bone marrow cells were harvested by flushing femurs and tibias of adult mice. A single-cell suspension was

obtained by passing cells through a pre-wet 70-µm cell strainer. Cells were differentiated into macrophages by culturing for 7 days at 37°C and 5 % CO_2 in 8 ml high glucose Dulbecco's modified eagle media (DMEM) supplemented with 10 % heat-inactivated fetal bovine serum (FBS, Gibco), 1 % penicillin/streptomycin (P/S) and 10 % supernatant derived from L929 fibroblasts (L929-condition media) as a source of macrophage colony-stimulating factor (Englen et al., 1995) in 10 cm non-tissue culture treated Petri dishes (ThermoFisher Scientific). BMDMs enrichment was confirmed by flow cytometry using F4/80 and CD11b as markers.

Real-time quantitative PCR (RT-qPCR)

Total RNA was extracted using miRVana™ isolation Kit (Life Technologies, AM1561) and RNA concentration and purity assessed using a NanoDrop ND-100 Spectrophotometer (Labtech). Total RNA (500 ng) was reverse transcribed using Quantities Reverse Transcription Kit (Qiagen, 205311). RT-qPCR was performed on a LightCycler 480 system (Roche) with Light cycler 480 Sybr Green I Master Mix (Roche, 04707516001) using Mm_Alox15_1_SG (QT00111034) and Mm_Pla2g4a_1_SG (QT00098259) (Qiagen) primers. Gene expression levels were normalized to 18S as housekeeping gene and relative RNA expression was calculated using the $2^{-\Delta\Delta CT}$ method.

Neutrophils isolation and apoptosis

Neutrophils were collected from bone marrow cells and blood in accordance with the Miltenyi Biotec neutrophil isolation kit protocol (130-097-658, Miltenyi Biotec). Briefly, bone marrow cells from femurs and tibias or 1 ml of blood obtained by cardiac puncture were incubated with 200 ul of biotinylated antibody cocktail (to recognise all cells apart from neutrophils) for 15 min at 4 °C. Cells were subsequently washed at 300g for 5 min, resuspended in FACs buffer and incubated with 100 ul of anti-biotin MicroBeads for 15 min at 4°C. Samples were then processed using pre-wet LS columns (130-042-401, Miltenyi Biotec) on a MACS magnet stand. Neutrophils enrichment was confirmed by flow cytometry using Ly6G and CD11b as markers. To induce apoptosis, $2x10^6$ neutrophils were incubated o/n in DMEM with 0.1% FBS at 37°C and 5% CO_2. Apoptosis induction was verified by flow cytometry using the Apotracker green (427402, Biolegend) V Zombie NIR™ Fixable Viability kit (423106, BioLegend), where Apotracker green -positive and Apotracker green/Zombie NIR™ positive cells were considered early and late apoptotic respectively. Apoptotic neutrophils were then washed with PBS and labelled with 1 µm CypHer5E NHS Ester (PA15401, Cytiva) in PBS for 30 min at room temperature, followed by washing twice with PBS.

***In vitro* macrophage efferocytosis**

Flow cytometry: WT and cKO BMDMs ($1x10^5$) were seeded in 24 well plates, followed by serum-starvation (1% FBS) for 16 h. Macrophages were treated with 100 nM MaR1 (10878, Cayman Chem), 5 nM activ-αMerTK antibody or vehicle (0.05% v/v EtOH or PBS) for 30 min. Apoptotic CypHer5E-labeled Neutrophils were then added to the macrophages at 10:1 ratio of apoptotic neutrophils:macrophage. After 3 h cells were detached using cell scrapers (CC7600-0220, Starlab), centrifuged at 300g for 10 min, and then resuspended in FACS buffer (0.5% BSA and 2 mM EDTA in PBS). The following antibodies were used: CD45.1 BV786 (103139, 5 µg/ml, clone 30-F1, BioLegend), F4/80 PE (12-4801-82, 0.25 µg/ml, clone BM8, ThermoFisher scientific), Ly6C APC (17-5932-82, 1 µg/ml, clone HK1.4, ThermoFisher scientific), CD11b BV421 (101236, 2 µg/ml, clone M1/70, BioLegend), MerTK BV605 (151517, 0.5 µg/ml, clone 2B10C42, BioLegend). Samples were run using LSRFortessa cell analyser (BD Bioscience) and analysed using FlowJo software (v10.1, BD Bioscience). All gating strategies were generated by using Fluorescent Minus One (FMO) controls and efferocytosis was quantified as % of $CypherNHS^+F4/80^+$ macrophages and BMDMs treated with unstained apoptotic neutrophils were used as control. Immunohistochemistry: 2 x 10^5 BMDM cells were placed over a coverslip and allocated in the incubator for adhesion. Cells were treated with the MerTK activating antibody (5 nM) for 30 min. Apoptotic CypHer5E-labeled Neutrophils were then added to BMDMs at 10:1 ratio of apoptotic neutrophils:macrophage. After 3 h of incubation of BMDM together with the apoptotic neutrophils, cells were washed and fixed in cold 4 % PFA (4 °C, 30 min). After fixation, cells were washed with PBS and then blocked in PBS with 0.1 % Triton and 0.2 % BSA (T-PBS; for intracellular staining) for 30 min at room temperature. Following blocking, cells were incubated with primary specific antibodies against F4/80 (ab111101, 5 µg/ml, Abcam) and MerTK (14-5751-82, 100 ng/ml, Molecular Probes Invitrogen) in T-PBS + 0.2 % BSA overnight at 4 °C. Cells were washed and incubated with secondary antibody Alexa Fluor 488 anti-rat (A21208, 1 µg/ml, Molecular Probes Invitrogen) or Alexa Fluor 647 anti-rabbit (A32795, 1 µg/ml, Molecular Probes Invitrogen) in T-PBS + 0.2 % BSA for 1 h at RT. Cells were then mounted on glass coverslips using Fluoroshield Histology Mounting Medium with DAPI (Sigma-Aldrich) and visualised under microscope (Zeiss LSM400 Imaging System). Tile scan images were acquired (4676 x 4676 µm), and quantification of total Cypher+ cells and F4/80+ was performed in a blind manner using the plugin Image-based Tool for Counting Nuclei for automated counting in ImageJ (version 1.45g).

DRG neurons in culture

DRGs were dissociated using 3 mg/ml Dispase (04942078001, Roche), 0.1% collagenase (C0773, Sigma-Aldrich), and 200 U/ml DNase I (10104159001, Sigma-Aldrich) in F-12 medium (11320033, Gibco). DRGs were subsequently triturated, and cell suspensions centrifuged at 300xg for 5 min. Pellets were resuspended in fresh DRG medium and plated on glass coverslips precoated with poly-L-ornithine (A-004-M, 100 µg/ml; Sigma-Aldrich) and laminin (11243217001, 40 µg/ml; Roche). Cultures (10,000 cells/well) were incubated at 37°C for 24 h, and then incubated with either vehicle (CON) or 1 µM capsaicin (CAPS) for 3 h in presence and absence of 5 µM GW4896 (D1692, Merck). Culture media was collected to isolate extracellular vesicles.

Extracellular vesicles isolation and analysis

Supernatants collected from cultured DRG neurons were centrifuged at 13,000xg for 2 min for removal of apoptotic bodies and cell debris. Supernatants were then ultracentrifuged at 100,000xg for 1 h at 4°C and resuspended in 1 ml of filtered sterile PBS. For nanoparticle tracking analysis, approximately 0.5 ml of extracellular vesicles (EV) preparations (between 10^6 to 10^8 vesicles) were loaded onto the Nanosight NS300 with 488 nm scatter laser and high sensitivity camera (Malvern Instruments Ltd., Malvern, UK); five videos of 90 s each were acquired for each sample. Data analysis was performed with NTA2.1 software (Nanosight, Malvern, UK). Analysis' software settings were the following, Detection Threshold: 5–10; Blur: auto; Minimum expected particle size: 20 nm. For arachidonic acid (AA) quantification, EV were resuspended in 100 µl of ELISA buffer and gently sonicated for 20s on ice. The EV suspension was then centrifuged at 14,000xg for 2 min to eliminate any debris and stored at -80˚C for quantification of (AA) by ELISA. For ImageStream analysis, EV were collected and analysed using ImageStream™, as previously described (9). EV were incubated with 50 µM BODIPY maleimide fluorescein (A-5770, Life Technologies) for 10 min on ice and acquired as such or after labelling with either 2 µg/ml anti-CD9-APC (124811, clone MZ3, Biolegend) or 2 µg/ml anti-CD63-PE (143903, clone NVG-2, Biolegend). Fluorescence minus one (FMO) controls were used for gating all protein antigen-positive events. Approximately 20,000 events were acquired per sample. EV samples were run on the ImageStream under slow-speed flow and ×60 magnification, with the 658 nm laser set at 200 mW and the side scatter at 70 mW. Data are expressed as EV/ml.

For Transmission Electron microscopy (TEM) analysis, TEM copper grids (400 mesh, Agar Scientific, Essex, UK) were pre-coated with 1% formvar (Agar Scientific, Essex, UK) solution made in chloroform. EV suspension (1 × 10^6/ml in 10 µl) was pipetted directly onto TEM grids; after 30 min, the TEM grids were washed with PBS, and fixed in 1% glutaraldehyde in PBS for 5 min. Thereafter, grids were stained in UA-Zero EM stain (Agar Scientific, Essex, UK) for 1 min. A JEOL 1400+ TEM (Tokyo, JPN) equipped with an AMT XR16 CCD camera (AMT, Massachusetts, USA) was used to acquire images.

Stimulation of BMDMs with EV

WT or cKO BMDMs (1x10^6) were plated in 12 well plates. In a first set of experiments cells were incubated for 3 h with EV isolated from DRG neuron media following incubation of either capsaicin (CAPS EV) or vehicle (CON EV) in presence and absence of 5 µM GW4869 (number of EV: CON EV, 7.34x10^5; CON + GW EV, 5.67x10^5; Caps EV, 1.58x0^6 EV; Caps + GW EV, 6.98x10^5). In a second set of experiments WT and cKO BMDMs were pretreated for 30 min with 100 µM of 5-LOX selective inhibitor Zileuton (10006967, Cayman chemical) prior stimulation for 3 h with EV isolated from DRG neuron media following incubation with either capsaicin (CAPS EV) or vehicle (CON EV) (number of EV: CON EV, 7.34x10^5; Caps EV: 1.58x0^6 EV). Supernatants from both experiments were centrifuged to deplete EV and kept for ELISA quantification of LTB_4.

Western blotting

WT and cKO BMDMs (2x10^6) were lysed in RIPA buffer (R0278, Sigma-Aldrich) supplemented with Halt phosphatase and protease cocktail inhibitor (78440, ThermoFisher scientific). Samples were loaded on a 10% w/v sodium dodecyl sulphate polyacrylamide tris–glycine gel (4561035, BioRad), transferred on PVDF membranes, and analysed by immunoblotting with 5-Lipoxygenase Antibody (NB110-58748, 1:1000, Biotechne) and GAPDH (ab8245, 1:2000, Abcam).

Tissue preparation for lipid mediator ELISAs

L3-L5 DRGs, paws and spinal cord dorsal horn were homogenised in 500 µl of ice-cold methanol, samples were then kept at -20˚C for 45 min and centrifuged at 1000xg for 5 min to precipitate proteins. Protein fraction was resuspended in 100 µl of PBS to determine protein concentrations by BCA assay (23225, ThermoFisher scientific) or run ELISA assays. Collected supernatants were evaporated at 37˚C under vacuum by using concentrator plus (Eppendorf, UK) and resuspended in 100 µL of ELISA diluent provided by manufacturer.

ELISA

Quantification of MaR1 in DRG, paw and dorsal horn homogenates, LTB_4, MaR1, PGE_2, LXA_4 and $CXCL_1$ in DRG extracts, LTB_4 in BMDMs supernatants following removal of EV, and AA in sonicated

EV, were measured by enzyme immunoassay kits (LTB_4, 10009292, MaR1, 501150, PGE_2, 514531, LXA_4, 590410, Cayman chemical; AA, ab287798, $CXCL_1$, ab190805, Abcam). DRG, paw and dorsal horn lipid mediator concentrations were normalised by the total content of proteins measured by BCA assay (23225, ThermoFisher scientific).

Statistical analyses

All data is presented as means ± SEM, with n representing either the number of mice used or the number of biological replicates (samples) obtained using different mice on different days. Sample size is reported in the figure legends and was determined according to previous internal data/publications and power analysis. Differences between means were considered statistically significant when $p<0.05$. Statistical analyses for behavioural and immunohistochemical data was performed using GraphPad Prism (v8.3.0; GraphPad Software, USA). For comparisons between two groups, an unpaired Student's t-test was used. For multiple group comparisons, one-way ANOVA followed by post hoc Tukey test was applied if more than two groups were involved, or two-way repeated measures ANOVA followed by post hoc Tukey test for behavioural testing, clinical scoring, and Western immunoblotting.

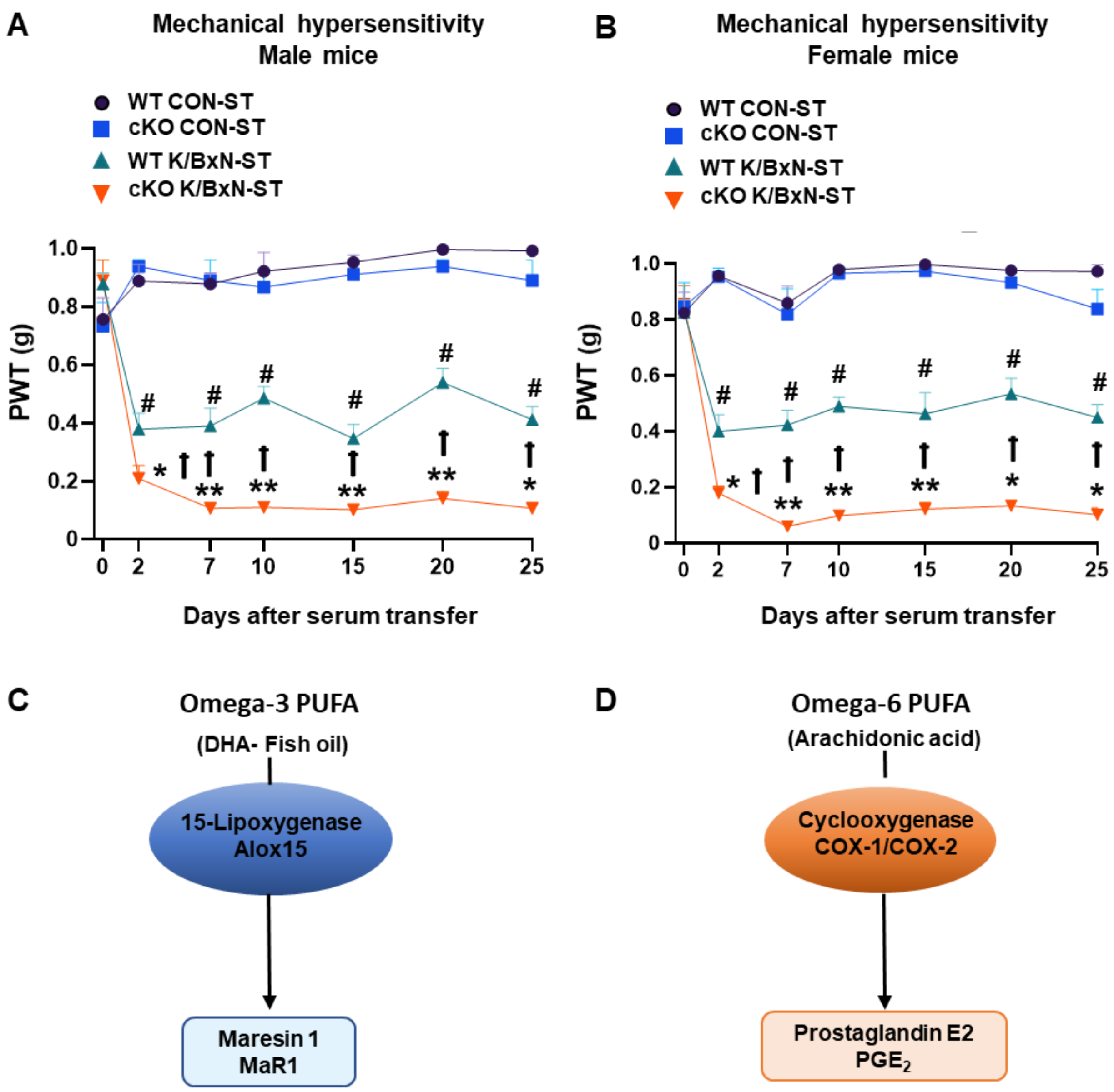

Fig. S1. Exacerbation of K/BxN serum transfer associated allodynia in Cx3cr1^{cre}Alox15^{floxed} male and female mice. (A, B) Hind paw mechanical hypersensitivity in male and female mice (allodynia, PWT; paw withdrawal thresholds). Data is mean ± SEM, n = 6 mice per group. $^*p < 0.05$, $^{**}p < 0.01$ cKO K/BxN-ST vs same-day CON-ST; $^{\#}p < 0.05$ WT K/BxN-ST vs same-day CON-ST; $^{\dagger}p < 0.05$, $^{\dagger\dagger}p < 0.01$ cKO K/BxN-ST vs same-day WT K/BxN-ST, two-way RM ANOVA followed by Tukey's multiple comparisons test. (C) Maresin 1 is biosynthesised from Omega 3 poly-unsaturated fatty acids (Docosahexaenoic Acid; DHA) by 15-Lipoxygenase enzyme. (D) Prostaglandin E2 is biosynthesised from Omega 6 poly-unsaturated fatty acids (arachidonic acid) by Cyclooxygenases 1 and 2 enzymes.

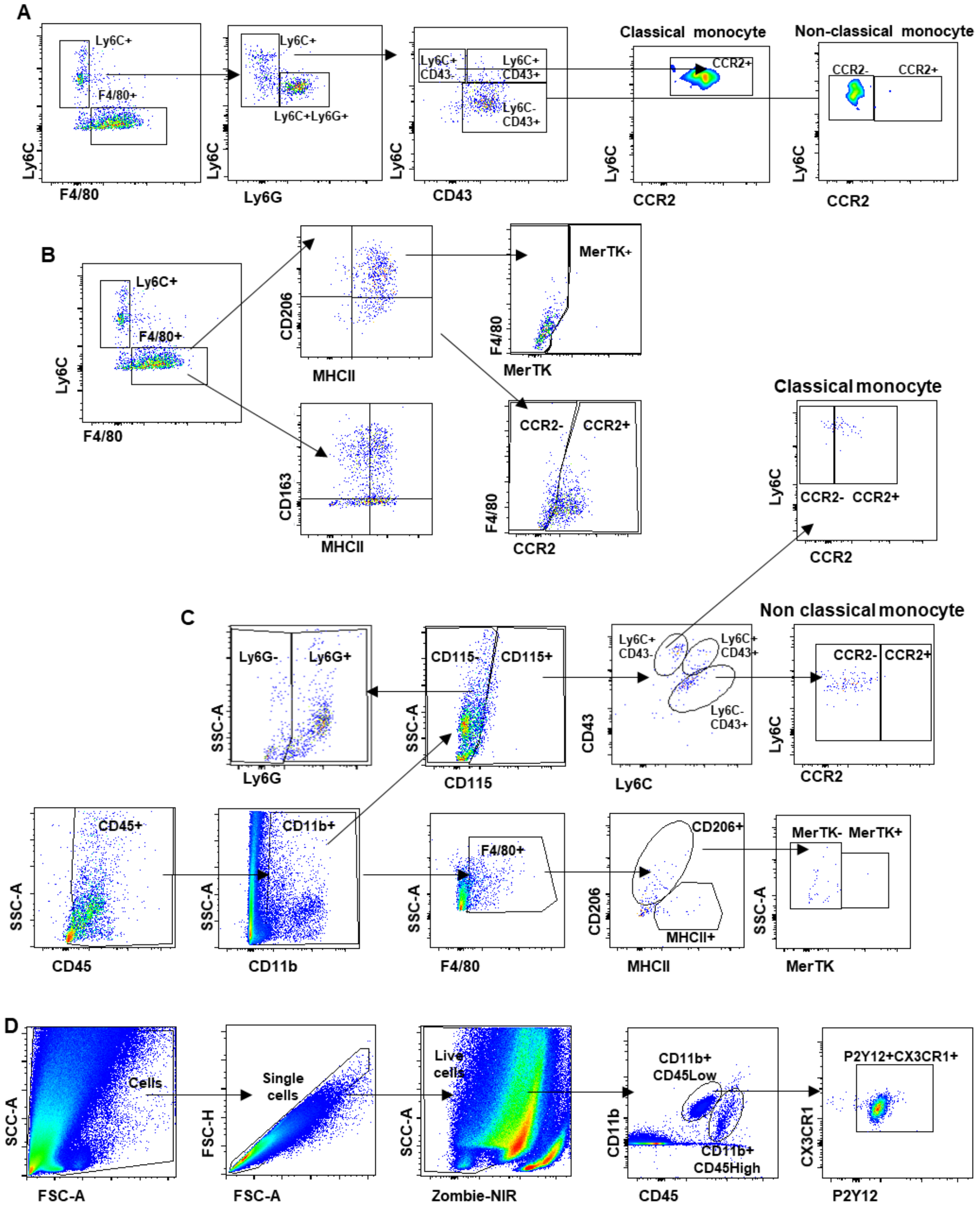

Fig. S2. Flow cytometry gating strategy for DRG, paw and microglia. Gating strategies were adapted from previous publication (Oggero et al, 2022; Ref.4). (A) Representative scatter plots for DRG: non-classical monocytes were gated as CD45^{+}CD11b^{+}F4/80^{-}Ly6G^{-}CD43^{+}Ly6C^{low}CCR2^{-}, classical monocytes as CD45^{+}CD11b^{+}Ly6G^{-}CD43^{+}F4/80^{-}Ly6C^{high}CCR2^{+} and neutrophils as CD45^{+}CD11b^{+}Ly6G^{+}Ly6C^{low}. (B) Representative scatter plots for DRG macrophages CD11b^{+}CD45^{+}Ly6C^{-}F4/80^{+} which were further analysed for M1-like (F4/80^{+}MHCII^{+}) and M2-like (F4/80^{+}CD206^{+}) phenotypes and perivascular macrophages (F4/80^{+}CD163^{+}). M1 and M2-like macrophages were further characterised for MerTK^{+} and CCR2^{+} expression. (C) Representative scatter plots for paw: non-classical monocytes were gated as CD45^{+}CD11b^{+}F4/80^{-}CD115^{+}CD43^{+}Ly6C^{low}CCR2^{-}, classical monocytes as CD45^{+}CD11b^{+}CD115^{+}CD43^{+}F4/80^{-}Ly6C^{high}CCR2^{+}, neutrophils as CD45^{+}CD11b^{+}CD115^{-}Ly6G^{+}Ly6C^{low} and macrophages as CD11b^{+}CD45^{+}F4/80^{+}. Macrophages were further analysed for M1-like (F4/80^{+}MHCII^{+}) and M2-like (F4/80^{+}CD206^{+}) phenotypes (D) Representative scatter plots of gating strategy for microglial cells (CX3CR1^{+}P2Y12^{+} cells).

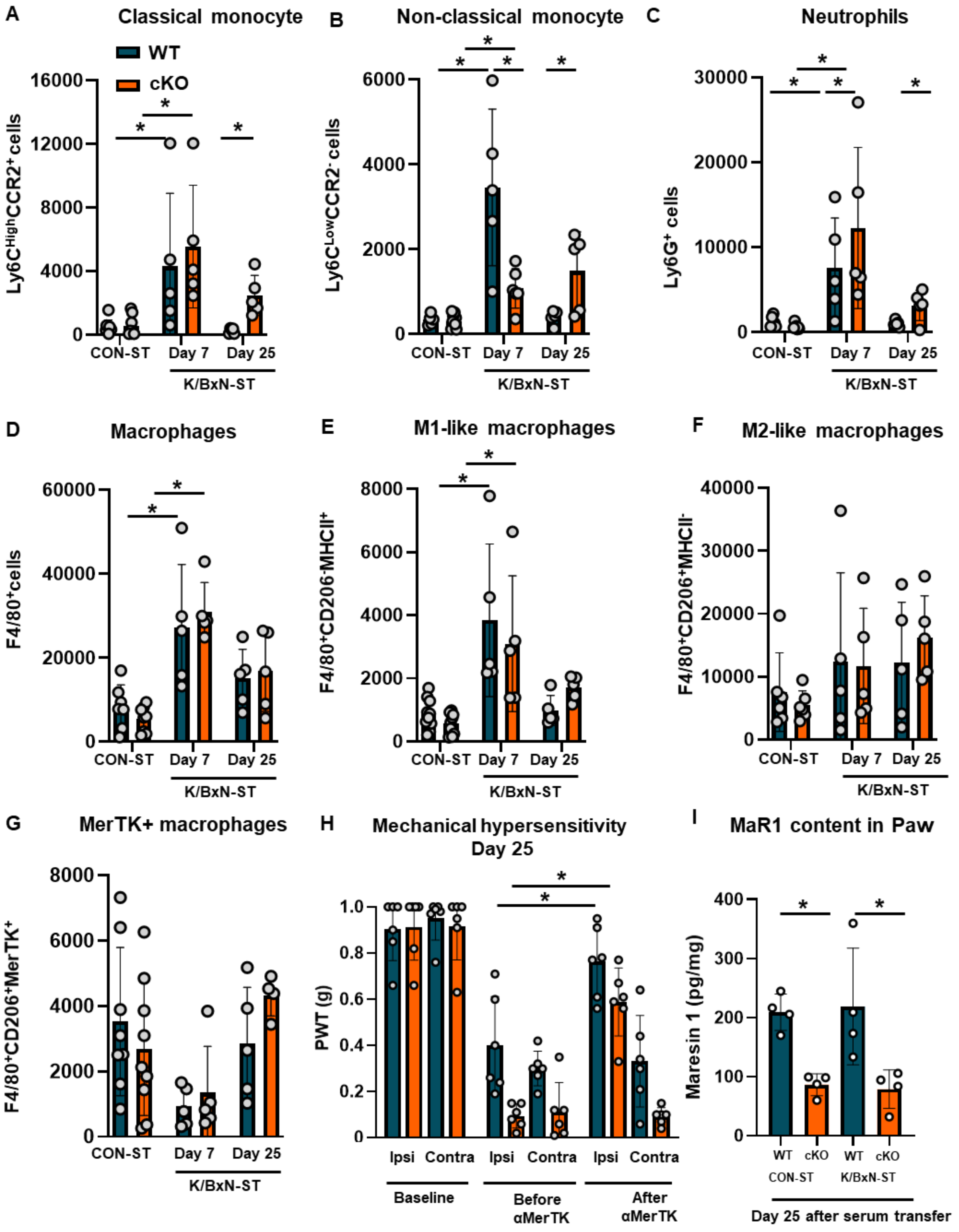

Fig. S3. Monocyte, neutrophil and macrophage infiltration in paw at day 7- and 25- K/BxN-ST. (A,B) Quantification of paw classical and non-classical monocytes, (C) neutrophils and (D) $CD45^+CD11b^+F4/80^+$ macrophage absolute numbers. (E) Quantification of paw $CD206^-MHCII^+$, (F) $CD206^+MHCII^-$, (G) $CD206^+MerTK^+$ macrophage absolute numbers. (H) Intraplantar injection of activating MerTK antibody at day 25- K/BxN-ST (activ-αMerTK; 100 pmol/mouse) reversed mechanical hypersensitivity in WT and cKO ipsilateral, but not contralateral to the injection site. (I) Quantification of Maresin 1 (MaR1) contents in paw at day 25- K/BxN-ST. Data is mean ± SEM. n = 6-12 biological replicates. *$p < 0.05$, one-way ANOVA followed by Tukey's multiple-comparison test.

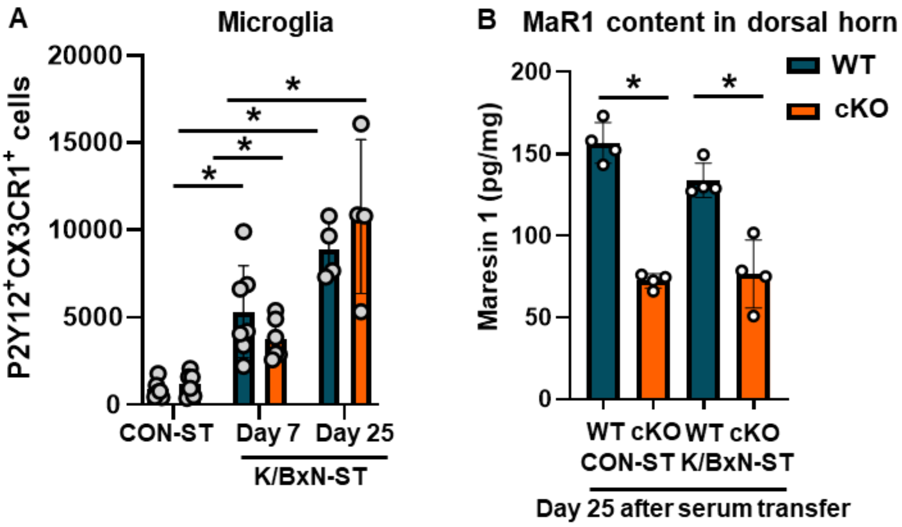

Fig. S4. Microglia activation at day 7 and day 25 after K/BxN serum transfer. (A) Quantification of spinal cord dorsal horn microglia absolute numbers. (B) Quantification of Maresin 1 (MaR1) content in spinal cord dorsal horn. Data is mean ± SEM. n = 4-7 biological replicates. *$p < 0.05$, one-way ANOVA followed by Tukey's multiple-comparison test.

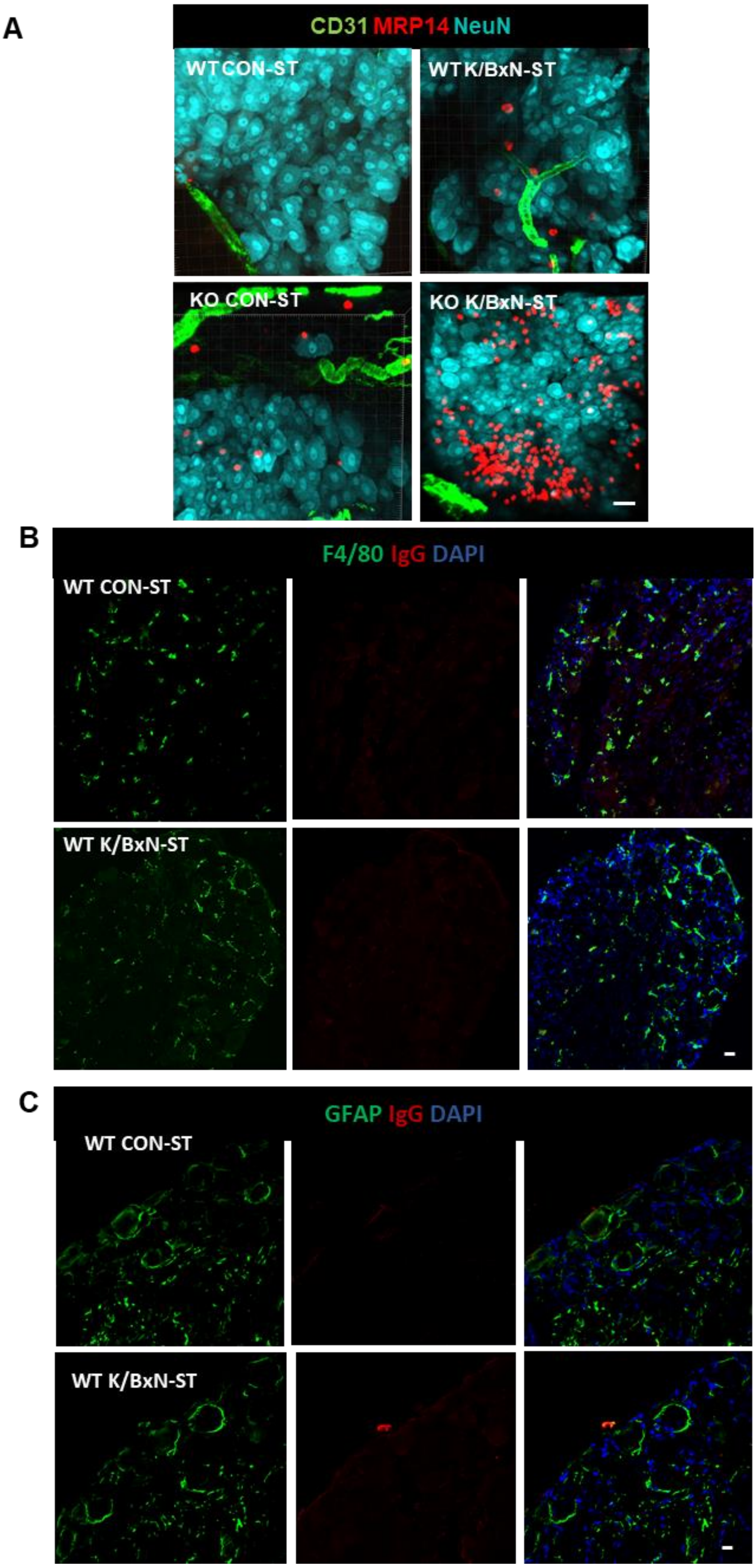
A
CD31 MRP14 NeuN
WT CON-ST
WT K/BxN-ST
KO CON-ST
KO K/BxN-ST
B
F4/80 IgG DAPI
WT CON-ST
WT K/BxN-ST
C
GFAP IgG DAPI
WT CON-ST
WT K/BxN-ST

Fig. S5. Neutrophils are not located in blood vessels at day-7 K/BxN-ST and IgG are not detected in macrophages and satellite glial cells at day-3 K/BxN-ST in WT and cKO. (A) 3D representative confocal images of L4 DRG whole mounts to visualise neurons (NeuN), blood vessels (CD31) and neutrophils (MRP14) in WT and cKO DRG at day 7-K/BxN ST. Scale bar is 30 µm.(B) Representative confocal images of L4 DRG to visualize macrophages (F4/80), and IgG. (C) Representative confocal images of L4 DRG to visualize satellite cell (GFAP) and IgG. Scale bar is 20 µm.

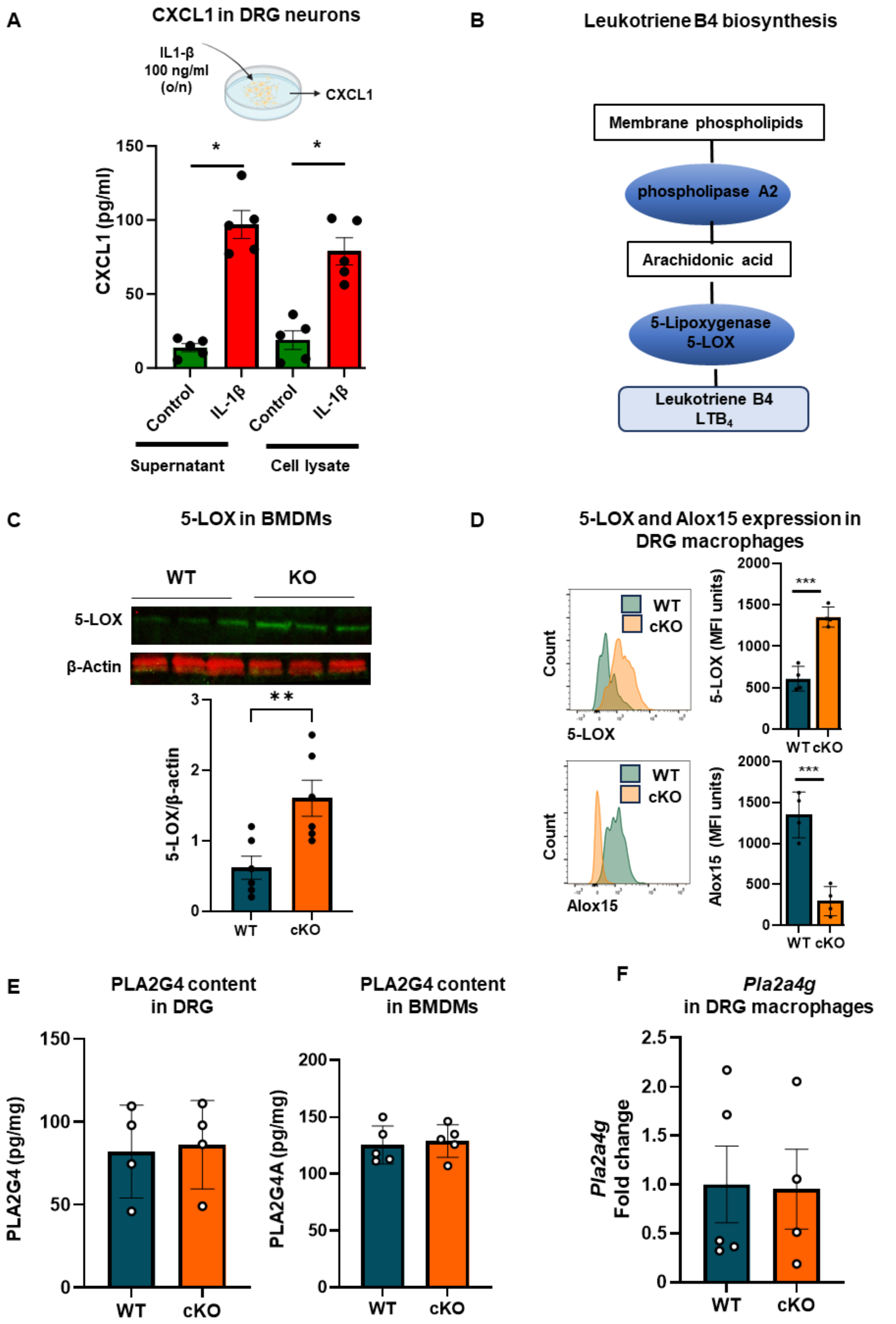

Fig. S6. IL-1β induces CXCL1 up-regulation in DRG neurons and silencing Alox15 in CX3CR1-expressing macrophages induces expression of 5-LOX enzyme but not PLA2G4. (A) Quantification of CXCL1 levels in DRG neurons and culture media after overnight (o/n) incubation with IL-1β (100 ng/ml). (B) Phospholipase A2 releases arachidonic acid from membrane phospholipids. The liberated arachidonic acid is converted in leukotriene B4 through the action of 5-LOX. (C) Representative Western blot and quantification of 5-LOX protein levels in BMDMs. (D) Representative histograms and quantification of 5-LOX and Alox15 expression, mean of fluorescence intensity (MFI) units in $CD45^{+}CD11b^{+}F4/80^{+}$ macrophages isolated from L3-L5 DRG. (E) Quantification of PLA2G4 content in WT and cKO DRG homogenates and BMDMs. (F) *Pla2g4a* fold change in WT and cKO DRG macrophages. Data is mean ± SEM., n = 4-6 biological replicates. *p< 0.05, **p < 0.01. Unpaired, 2-tailed Student's t test.

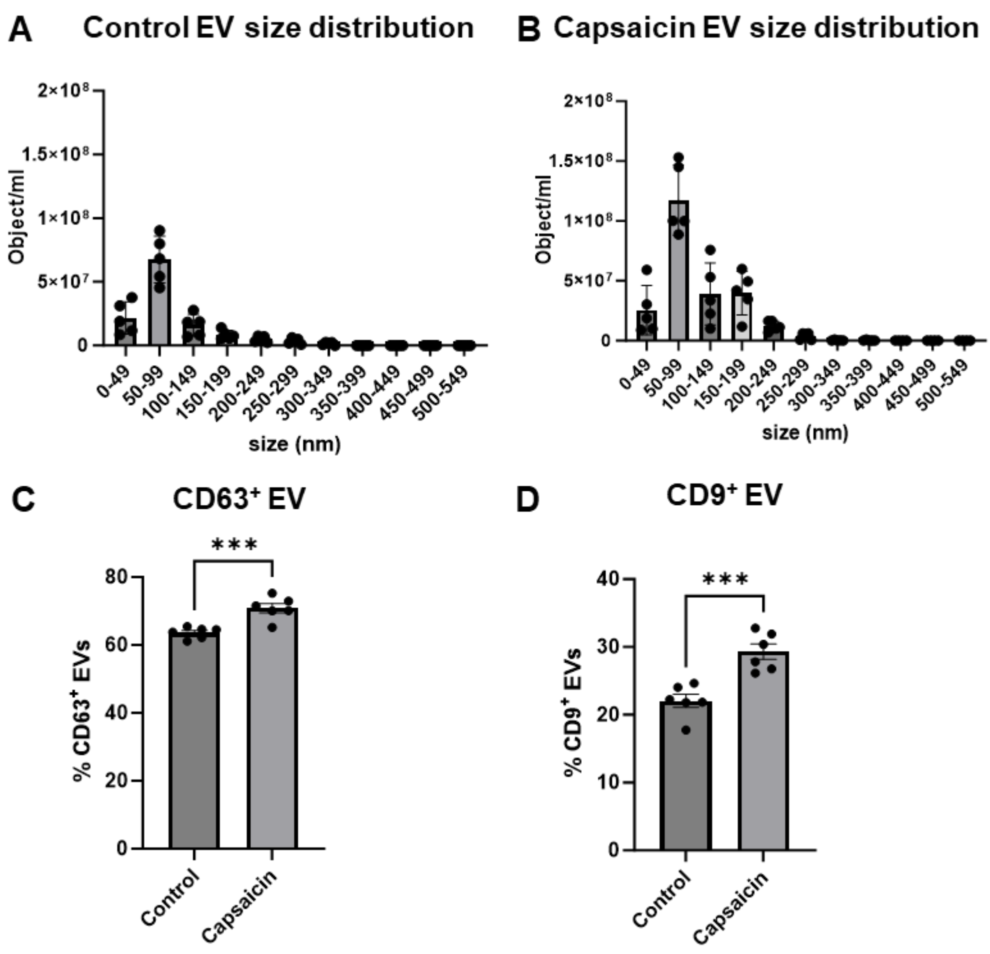

Fig. S7. Neuron-derived EV are exosomes and express tetraspanin markers CD9 and CD63. Bar charts represent Nanoparticle tracking size distribution of EV isolated from (A) vehicle or (B) capsaicin (1 µM; 3h) stimulated DRG neurons. Bar charts represent Imagestream analysis of (C) $CD63^+$ and (D) $CD9^+$ EV percentages in samples obtained from vehicle or (C) capsaicin (1 µM; 3 h) stimulated DRG neurons. Data is mean ± SEM., n = 5, 6 biological replicates for each group. ***$p < 0.001$. Unpaired, 2-tailed Student's t test.

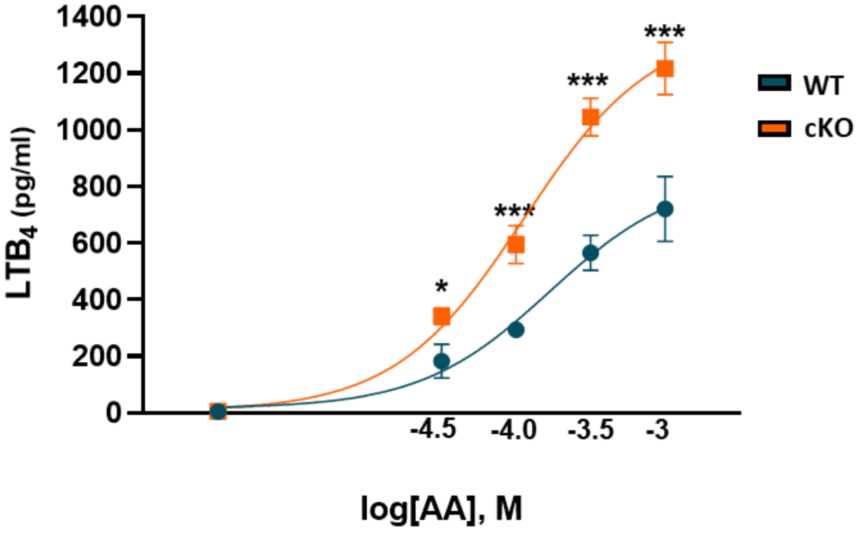

Fig. S8. In BMDMs arachidonic acid (AA) is substrate for leukotriene B4 production. Concentration-response curves of AA mediated leukotriene B4 (LTB_4) production. Data is mean ± SEM., n = 4 biological replicates for each group. *p<0.05, ***p < 0.001 Unpaired, 2-tailed Student's t test.

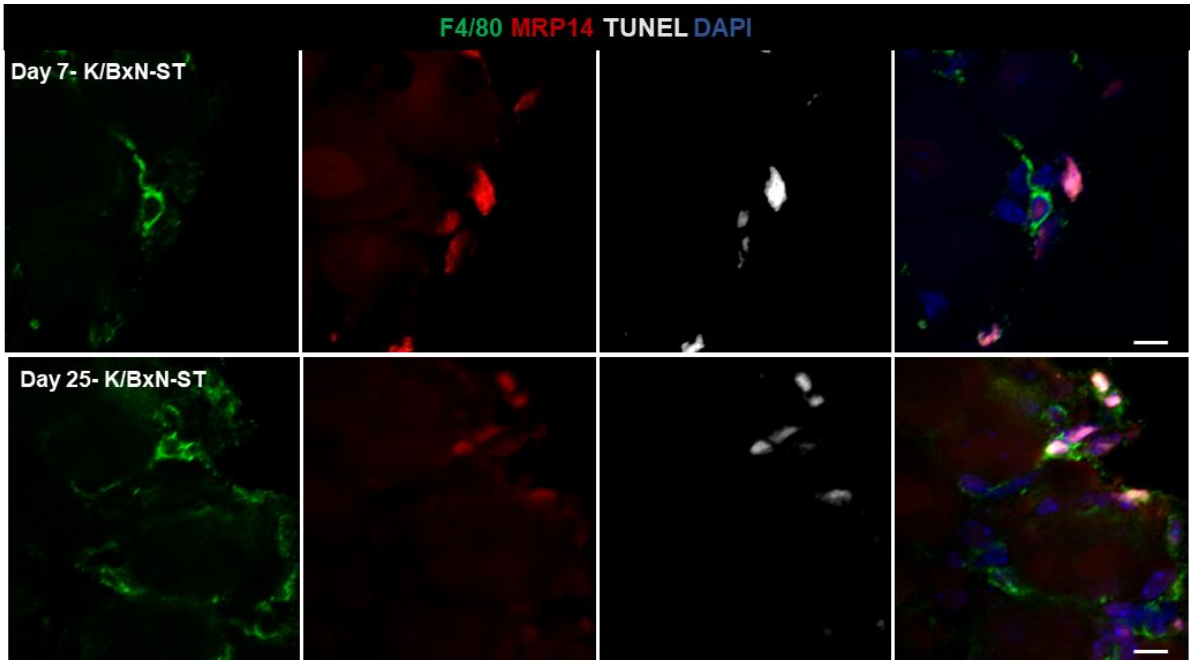

Fig. S9. DRG macrophage efferocytosis of apoptotic neutrophils. (A, B) Representative confocal images of L4 DRG to visualize macrophage (F4/80) efferocytosis of apoptotic neutrophils (Tunel$^+$, MRP14$^+$) in WT DRG at Day 7- and Day 25- K/BxN-ST. Scale bar is 20 µm.

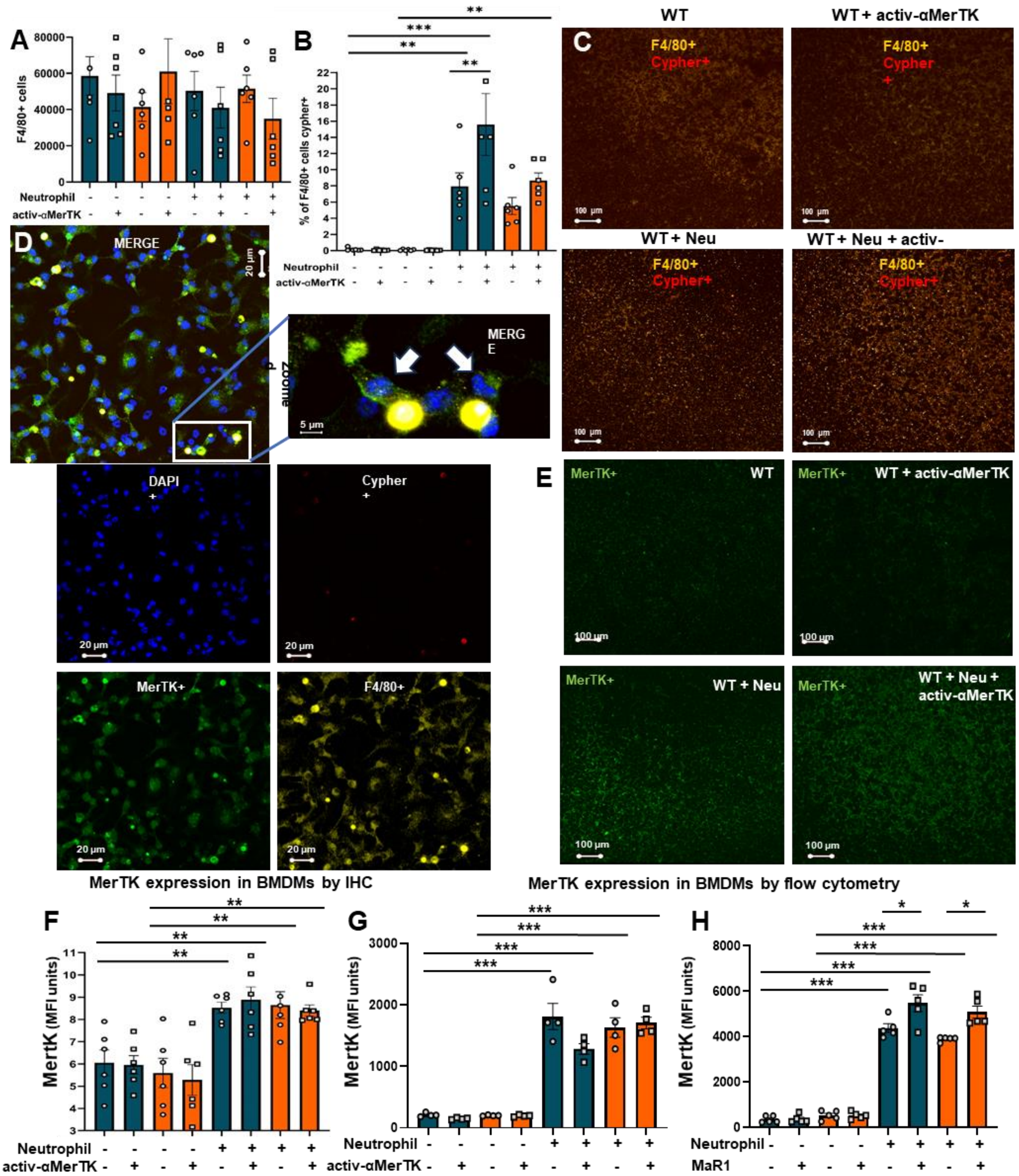

Fig. S10. Additional characterisation of efferocytosis and MER Proto-Oncogene, Tyrosine Kinase (MerTK) expression in BMDMs. (A) Quantification of BMDM cells (F4/80^{+}) in the different groups. (B) Quantification and (C) representative image of F4/80^{+}/CypherNHS^{+} BMDMs in presence and absence of activating MerTK antibody (activ- αMerTK; 5 nM). (D) Representative image of F4/80+ macrophage engulfing neutrophil. White arrows point BMDM cells (F4/80^{+} and MerTK^{+}) containing neutrophils (Cypher^{+}). (E,F) Immunostaining of MerTK expression in F4/80^{+} BMDMs in presence and absence of activ- αMerTK (5 nM). (G) Quantification of MerTK expression (MFI) in F4/80^{+} BMDMs in presence and in absence activ-αMerTK. and (H) MaR1. Data is mean ± SEM. n = 5, 6 biological replicates. *p < 0.05, **p < 0.01, ***p< 0.001; one-way ANOVA followed by Newman-Keuls test.

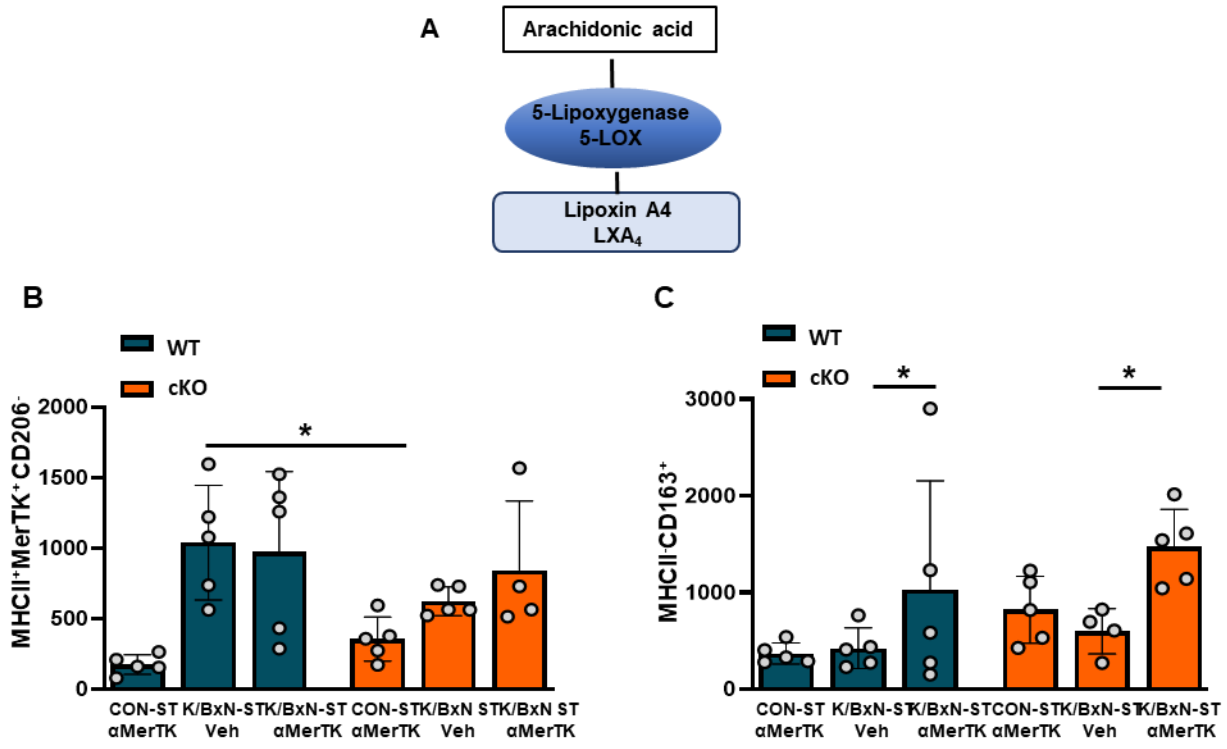

Fig. S11. Additional characterisation of MerTK and CD163 expression in DRG macrophages. (A) arachidonic acid is converted into Lipoxin A4 by 5-LOX enzyme. (B) Quantification of L3-L5 DRG F4/80$^+$MHCII$^+$MerTK$^+$CD206$^-$ macrophage absolute numbers at day 25- K/BxN ST, 48 hours after i.t. delivery of activ- αMerTK (αMerTK). (C) Quantification of L3-L5 DRG MHCII$^-$CD163$^+$ macrophage absolute numbers, 48 hours after i.t. delivery of activ- αMerTK. Data is mean ± SEM. n = 5 biological replicates. *$p < 0.05$, one-way ANOVA followed by Tukey's multiple-comparison test.

GRAPHICAL ABSTRACT

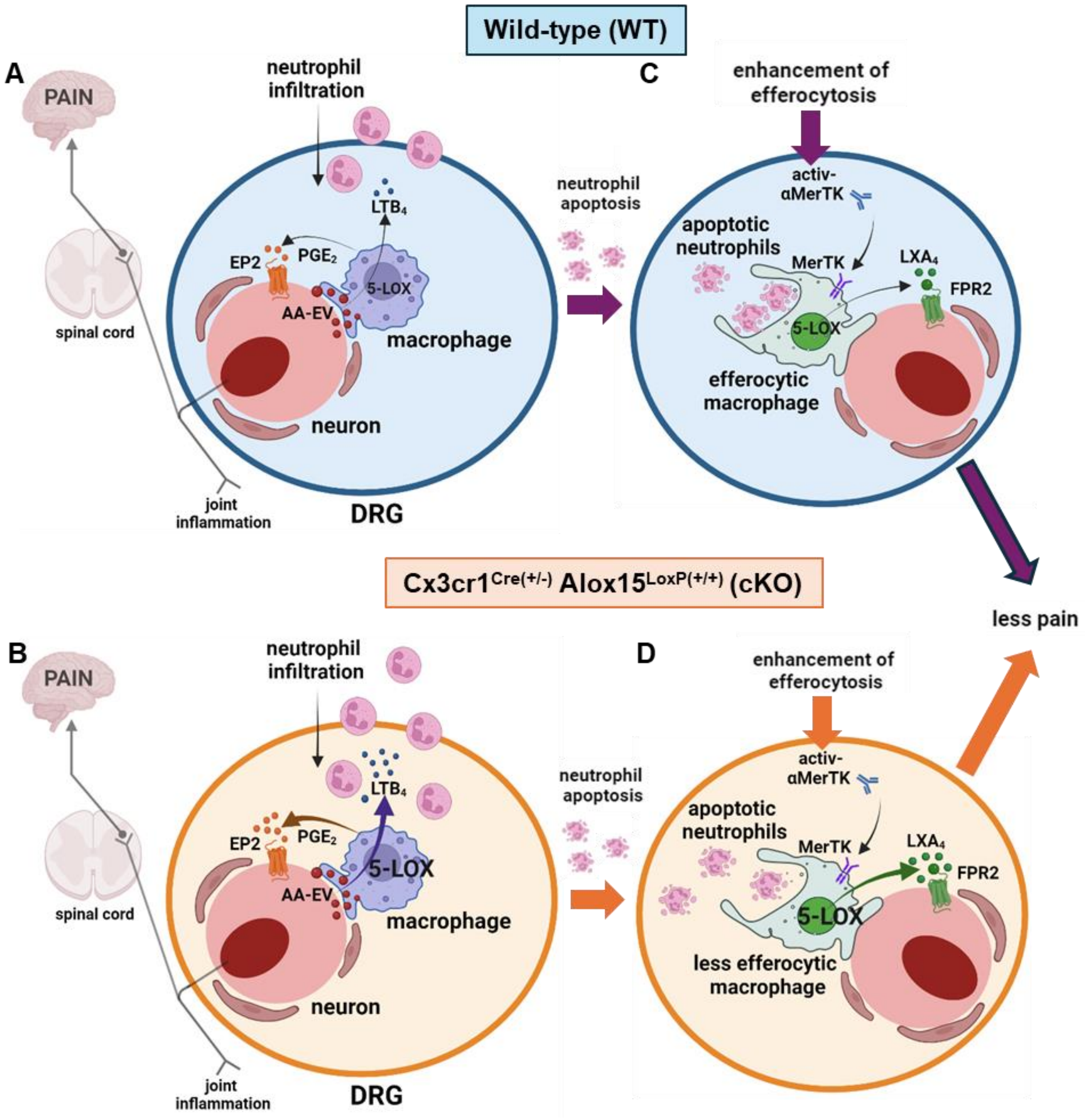

Fig. S12. Enhancement of pro-resolving macrophages attenuates persistent inflammatory pain. (A) DRG neuron activity results in release of extracellular vesicles, which provide macrophages with arachidonic acid (AA) for 5-LOX-mediated production of chemoattractant LTB_4 that promotes neutrophil infiltration. In addition, macrophages produce lipid mediators PGE_2 that promotes nociceptive signalling via neuronal EP2 receptor activation. (B) In Alox5 conditional knockout (cKO) DRG, higher levels of 5-LOX result in higher production of LTB_4 and larger neutrophil infiltration. (C) Afterwards, spontaneous neutrophil apoptosis promotes macrophage efferocytosis that can be enhanced by MerTK activating antibody to produce lipid mediator LXA_4 that attenuates nociceptive signalling through activation of neuronal FPR2 receptor. (D) Spontaneous neutrophil apoptosis promotes macrophage efferocytosis that is less effective in cKO. However, in both WT and cKO, macrophage efferocytosis can be enhanced by MerTK activating antibody to produce lipid mediator lipoxin A4 (LXA4) which attenuates nociceptive signalling through activation of formyl peptide receptor 2 (FPR2). In cKO, LXA4 production by 5-LOX further increases levels of this anti-nociceptive lipid mediator.

Table S1. Flow cytometry Paw				
**antibodies**	**clone**	**cat number**	**company**	**concentration**
CD45 BV450	30-F11	560501	BD Biosciences	0.5 µg/ml
CD11b PE-Cy7	M1/70	25-0112-81	ThermoFisher scientific	0.2 ug/ml
CD115 APC	AFS98	135509	Biolegend	2 µg/ml
Ly6C PerCP Cy5.5	HK1.4	45-5932-82	ThermoFisher scientific	1 µg/ml
Ly6G PE	1A8	551460	BD Bioscience	0.5 µg/ml
CD43 BV510	S7	563206	BD Bioscience	1 mg/ml
MHCII AF700	M5/114	107622	Biolegend	1.25 µg/ml
F4/80 BV650	BM8	123149	ThermoFisher scientific	2 µg/ml
CCR2 BV711	475301	FAB5538P	BD Biosciences	0.5 µg/ml
CD206 BV605	C068C2	141721	Biolegend	3 µg/ml
CX3CR1 AF488	149021	SA011F11	Biolegend	1 µg/ml
MerTK BV605	151517	2B10C42	Biolegend	0.5 µg/ml

Table S1. List of flow cytometry antibodies used for leukocyte characterisation in paws

Table S2. Flow cytometry DRG				
**antibodies**	**clone**	**cat number**	**company**	**concentration**
CD45.1 BV786	30-F11	103139	Biolegend	0.5 µg/ml
CD11b BV421	M1/70	101236	Biolegend	1 ug/ml
CD206 PE-Cy7	C068C2	141720	Biolegend	5 µg/ml
Ly6C APC	HK1.4	17-5932-82	ThermoFisher scientific	1 µg/ml
Ly6G AF700	1A8	127622	Biolegend	1 µg/ml
CD43 BUV737	S7	612840	BD Bioscience	1 mg/ml
MHCII PerCP-Cy5.5	N418	116416	Biolegend	2 µg/ml
F4/80 PE	BM8	12-4801-82	ThermoFisher scientific	0.25 µg/ml
CCR2 BV711	475301	FAB5538P	BD Biosciences	0.5 µg/ml
CD163 PE-eFluor	TNKUPJ	61-1631-82	ThermoFisher scientific	1 µg/ml
CX3CR1 AF488	149021	SA011F11	Biolegend	1 µg/ml
MerTK BV605	151517	2B10C42	Biolegend	0.5 µg/ml
P2YR12 PE	S16007D	848004	Biolegend	0.5 µg/ml

Table S2. List of flow cytometry antibodies used for leukocyte characterisation in DRG and microglia characterisation in spinal cord dorsal horn.

SI Reference

Reference

1. D. S. Koenis, R. de Matteis, V. Rajeeve, P. Cutillas, J. Dalli, Efferocyte-Derived MCTRs Metabolically Prime Macrophages for Continual Efferocytosis via Rac1-Mediated Activation of Glycolysis. *Adv Sci (Weinh)* **11**, e2304690 (2024).
2. L. V. Norling *et al.*, Proresolving and cartilage-protective actions of resolvin D1 in inflammatory arthritis. *JCI Insight* **1**, e85922 (2016).
3. B. L. Allen *et al.*, Imbalance of proresolving lipid mediators in persistent allodynia dissociated from signs of clinical arthritis. *Pain* **161**, 2155-2166 (2020).
4. S. Oggero *et al.*, Dorsal root ganglia CX3CR1 expressing monocytes/macrophages contribute to arthritis pain. *Brain Behav Immun* **106**, 289-306 (2022).
5. M. L. Bennett *et al.*, New tools for studying microglia in the mouse and human CNS. *Proc Natl Acad Sci U S A* **113**, E1738-1746 (2016).
6. G. Sideris-Lampretsas *et al.*, Galectin-3 activates spinal microglia to induce inflammatory nociception in wild type but not in mice modelling Alzheimer's disease. *Nat Commun* **14**, 3579 (2023).
7. F. M. Marim, T. N. Silveira, D. S. Lima, Jr., D. S. Zamboni, A method for generation of bone marrow-derived macrophages from cryopreserved mouse bone marrow cells. *PLoS One* **5**, e15263 (2010).
8. L. Zeboudj *et al.*, Silencing miR-21-5p in sensory neurons reverses neuropathic allodynia via activation of TGFB-related pathway in macrophages. *J Clin Invest* 10.1172/JCI164472 (2023).
9. R. Simeoli *et al.*, Exosomal cargo including microRNA regulates sensory neuron to macrophage communication after nerve trauma. *Nat Commun* **8**, 1778 (2017).
